# Response of soil microbial community diversity and structure to soybean-based intercropping and its effects on yield

**DOI:** 10.3389/fmicb.2025.1658783

**Published:** 2025-08-29

**Authors:** Xinyi Zhan, Yongwei Shu, Liu Guo, Xinyue Liu, Quan Zhao, Yuze Li, Taiwen Yong, Wenyu Yang

**Affiliations:** ^1^College of Agronomy, Sichuan Agricultural University, Chengdu, China; ^2^Sichuan Engineering Research Center for Crop Strip Intercropping System/Key Laboratory of Crop Ecophysiology and Farming System in Southwest, Ministry of Agriculture, Chengdu, China; ^3^College of Landscape Architecture, Sichuan Agricultural University, Chengdu, China

**Keywords:** intercropping, rhizosphere, microorganism, nitrogen fertilizer, yield

## Abstract

**Introduction:**

Soybean-based intercropping has emerged as a key sustainable agricultural practice, which enhances system productivity and improves soil health. Although numerous studies have investigated soybean yield under intercropping systems, the responses of soil microbial community traits and their associations with yield remain unclear.

**Methods:**

Based on 323 observations extracted from 89 peer-reviewed articles, we conducted a meta-analysis to investigate the responses of soil microbial community traits and crop yield to soybean-based intercropping.

**Results:**

Compared to monoculture, our analysis revealed that soybean-based intercropping did not significantly affect soybean yield (effect size = −0.020, 95% CI: −0.105 to 0.065), but significantly changed soil microbial communities. The practice significantly enhanced microbial community α-diversity indices, including Shannon (effect size = 0.036, 95% CI: 0.020 to 0.053), Chao1 (effect size = 0.034, 95% CI: 0.002 to 0.067), and richness (effect size = 0.102, 95% CI: 0.026 to 0.178), while also significantly altered microbial community structure (effect size = 0.741, 95% CI: 0.629 to 0.852). Random forest analysis identified nitrogen fertilization rate as the primary predictor of α-diversity indices. And nitrogen fertilization rate showed a significant negative correlation with Chao1 (*R*^2^ = 0.051, *p* = 0.079) and a marginally significant negative correlation with richness (*R*^2^ = 0.141, *p* = 0.018). Nitrogen fertilizer type significantly affected soil microbial α-diversity, with mixed nitrogen fertilizers demonstrating greater effects on both Chao1 (effect size = 0.111, 95% CI: 0.034 to 0.188) and richness (effect size = 0.192, 95% CI: 0.038 to 0.345) than mineral fertilizers. Intriguingly, while we only found a marginally significant positive correlation between Chao1 and soybean yield (*R*^2^ = 0.131, *p* = 0.082), yield advantages over monoculture emerged within a specific Shannon index range from 0.008 to 0.401. Given the lack of a direct association between nitrogen fertilization rate and soybean yield (*r* = 0.056, *p* > 0.05), its influence on soybean yield in soybean-based intercropping may mediated by the changes in microbial community diversity.

**Conclusion:**

Collectively, these findings reveal soil microbial responses to soybean-based intercropping and highlight the potential for optimizing microbial communities to enhance soil health and agricultural sustainability in intercropping systems.

## 1 Introduction

Intercropping refers to the cultivation practice of growing two or more crop species with similar growth periods simultaneously in the same field, with specific spatial arrangements. This system provides multiple agronomic benefits including suppressing weeds, reducing greenhouse gas emissions, and enhancing system productivity (Li et al., 2020). Soybean-based intercropping (SBI) represents one of the most prevalent crop combinations in agriculture, with its nitrogen fixation capacity and complementary nutrient use efficiency having been extensively studied (Lai et al., 2022). However, soybean is frequently considered as a secondary “intercrop” planted, primarily used for increasing yields of focus crops such as maize or sorghum (Liu et al., 2017). Consequently, soybean yield is commonly reported to be significantly lower than in monoculture (Yang et al., 2024). Current research presents a dichotomy in soybean yield responses to intercropping systems. In contrast, some studies have demonstrated yield improvements in intercropped soybean (Zhang et al., 2023), but the precise mechanisms underlying these divergent results remain unclear.

Soil microbial communities, comprising fungi and bacteria, perform numerous ecologically significant functions (Nemergut et al., 2014), including critical processes like nutrient cycling and nitrogen fixation that are essential for ecosystem functioning (Hungria et al., 2009). In nutrient-limited environments, these microorganisms become pivotal regulators of plant growth via nutrient mineralization and competitive acquisition (van der Heijden et al., 2008), also enhancing crop disease resistance and triggering plant immune responses by producing enzymes and antimicrobial compounds (Berendsen et al., 2012). Microbial diversity significantly influences soil microbial functionality (Fierer, 2017), with higher diversity promoting ecosystem stability (Maron et al., 2018), particularly for processes mediated by small pools of microbial species that are especially sensitive to diversity changes (Peter et al., 2011). As soil microbial diversity supports agroecosystem functions like plant productivity and decomposition through multiple mechanisms (Hungria et al., 2009), thereby affecting crop growth and ecosystem performance by modulating microbial functions. Recent studies on SBI have extensively investigated aboveground yield-enhancement mechanisms, such as the formation of distinct ecological niches between soybean and companion crops that improve light-use efficiency to achieve yield gains through the “dense planting effect” (Wang and Liu, 2023), while appropriate spatial arrangements can also enhance photosynthetic performance by increasing chlorophyll content and photosynthetic capacity in intercropped species (Hidayat et al., 2021). However, compared to studies on aboveground components, our understanding of how intercropping affects the belowground parts, typically soil microbial communities remains limited. As an important cultivation practice, intercropping requires comprehensive evaluation of soil microbial responses to clarify their functional roles in intercropping systems. While studies have demonstrated that alterations in bacterial community structure significantly influence crop yield (Liu et al., 2022), it remains unclear whether changes in microbial community traits are associated with soybean yield in SBI systems.

For intercropping systems, crops alter soil microenvironments through root exudate-mediated competition for soil nutrients, thereby altering soil microbial diversity (Wang et al., 2018). Compared to long-term soybean monoculture, the soybean-maize intercropping enhances rhizosphere microbial diversity while improving the complexity and stability of microbial interaction networks (Chang et al., 2022) Notably, intercropping exerts distinct effects on bacterial and fungal community diversity and structure (Xiao et al., 2023). For instance, soybean-maize intercropping increases both bacterial and fungal diversity, modifies soil community structure through interspecific interactions, and elevates the relative abundance of beneficial bacterial taxa involved in nutrient cycling (Guo et al., 2024). For SBI system, it has been documented that the increased rhizosphere microbial diversity was attributed to the enhancement of root exudation (Yong et al., 2012). Moreover, when intercropped with maize, soybean exudates more daidzein and genistein to reshape the rhizosphere rhizobia community structure and diversity, thus, the nodule nitrogen fixation and yield were improved (Lin et al., 2023).

However, some studies have reported to have no significant effects of intercropping on soil microbial diversity (Lu et al., 2025). Notably, some others even shown reduced rhizosphere microbial diversity. This may be attributed to the higher nutrient consumption caused by the more complex crop populations in intercropping systems compared with monoculture, especially the microorganisms are often at a disadvantage in competing with plants for nitrogen (Zhang et al., 2022). Furthermore, different intercropping systems alter the rhizosphere microenvironment, modifying critical factors such as soil temperature, moisture, and the availability of carbon, nitrogen, and mineral nutrients, potentially creating unfavorable conditions for microbial communities (Wu et al., 2019). The response of microbial diversity to SBI remains unclear, and its relationship with yield requires further investigation. Therefore, a systematic analysis is needed to elucidate the specific mechanisms through which SBI influences soil microbial diversity and, consequently, soybean yield.

Numerous studies have documented the impacts of intercropping on soil microbial community traits, including the changes in soil microbial functional diversity induced by green manure intercropping in tea plantations (Wang et al., 2023) and the increased soil nutrients content observed in maize-soybean intercropping compared to monocultures (Yu et al., 2025). Considering the vital role of microorganisms in intercropping systems, elucidating their functions in SBI, particularly their relationship with crop yield, becomes crucial for optimizing soybean production. To clarify how soil microbial communities respond to SBI and contribute to the yield, a comprehensive synthesis on the microbial community traits including α-diversity, β-diversity, and structure is essential. Here, based on 323 observations from 89 peer-reviewed articles, we aim to explore the responses of soil microbial community α-diversity (Shannon index, richness, Chao1), β-diversity, and structure to intercropping, analyze the influences of different management factors, sampling sites and environmental factors on these traits, and examine the relationships between them and soybean yield. We hypothesize that: (1) intercropping will significantly increase soil microbial α- and β-diversity by facilitating root interactions and improving soil environments; (2) management factors (e.g., nitrogen application, intercropping configuration) and environmental factors (e.g., pHi) will significantly affect changes in microbial community traits; (3) enhanced microbial diversity was beneficial to narrow the yield gap between intercropped and monocultured soybean.

## 2 Materials and methods

### 2.1 Data collection

To investigate the effects of SBI on soil microbial α-diversity, β-diversity, and structure, we conducted an extensive search of peer-reviewed articles in April 2024 across multiple databases including Web of Science and CNKI. The initial screening employed the following keyword combinations: “soybean “and “intercrop” and “microbial community” or “microbiome.” Articles were then selected for meta-analysis based on four criteria: (1) field-based studies with experimental designs allowing paired comparisons between SBI treatments; (2) reporting of at least one soil microbial community parameter (α-diversity, β-diversity, or structure); (3) treatment pairs established under identical ecosystem types, crop varieties, and soil conditions; and (4) for studies with multiple growing seasons or years of data, only the final year’s data were extracted for meta-analysis to minimize data non-independence. Following the PRISMA flowchart, we collected 323 observations from 89 peer-reviewed articles that reported effects of SBI on soil microbial communities (Supplementary Figure S1).

In addition to soil microbial community traits, we extracted supplementary information including sampling sites, experimental duration, fertilization regime, nitrogen application rate, soybean or non-soybean strip, and sampled soil compartments. Furthermore, other paired measurements including initial soil organic carbon (SOCi), initial total nitrogen (TNi), initial soil pH (pHi), and soybean yield were systematically recorded to analyze potential drivers of soil microbial community changes and establish their connections with agricultural productivity. To better account for density differences between intercropping and monoculture systems, the total relative planting density was employed as a standardized metric for comparison.


$$\mathrm{RDT}=\frac{d_{1,\mathrm{ic}}}{d_{1,\mathrm{sc}}}+\frac{d_{2,\mathrm{ic}}}{d_{2,\mathrm{sc}}}$$

where *d*_1,ic_, *d*_2,ic_ are densities of species 1 and 2, respectively, in the intercrop, and *d*_1,sc_ and *d*_2,sc_ are densities of species 1 and 2, respectively, in sole crops. Density of a crop species in an intercrop is defined as the number of plants per unit land area of the whole intercrop (i.e., including the area occupied by other species).

Specifically, the planting density difference between soybean intercropping and monoculture systems was quantified using the plant relative density total (pRDT) metric as a standardized measurement approach.


$$\mathrm{RDT}=\frac{d_{\mathrm{ic}}}{d_{\mathrm{sc}}}$$

where *d*_ic_, *d*_sc_ are densities of soybean in the intercrop or in sole crops, inseparately. Density of a crop species in an intercrop is defined as the number of plants per unit land area of the whole intercrop (i.e., including the area occupied by other species).

An RDT equal to 1 indicates replacement intercropping, and RDT > 1 indicates additive intercropping. Intercrops with RDT <1 were excluded from the database (Li et al., 2024).

### 2.2 Data analysis

All statistical analyses were performed using R version 3.6.0. The effect of SBI on soil microbial community traits (α-diversity, β-diversity, and structure) was estimated and calculated using the response ratio (RR), which is independent of the study design, as follows:

$$\text{lnRR}=\ln\frac{x_{t}}{x_{c}}=\ln x_{t}-\ln x_{c}$$

Among these, *x*_c_ and *x*_t_ represent the mean values of indicator *x* in the control (monoculture) and treatment (intercropping) groups, respectively.

In single studies, it is often necessary to estimate multiple variables simultaneously, and non-independence of data is a common phenomenon in meta-analyses (Noble et al., 2017). Therefore, we employed mixed-effects models to assess the effects of SBI on soil microbial community traits and other related parameters, thereby accounting for the nested structure and non-independence of observations. These models were constructed using the rma.mv() function in the “metafor” package, and overall effects along with 95% confidence intervals (CI) were calculated (Viechtbauer, 2010). The variance (*v*) of lnRR was calculated as follows:


$$v=\frac{s_{t}^{2}}{n_{t}x_{t}^{2}}+\frac{s_{c}^{2}}{n_{c}x_{c}^{2}}$$

In this formula, *n*_c_ and *n*_t_ represent the sample sizes for the control (monoculture) and treatment (intercropping), respectively, while *s*_c_ and *s*_t_ represent the standard deviations for the control (monoculture) and treatment (intercropping), respectively.

If the 95% CI for the overall effect size of the indicator does not cross the zero point on the x-axis, then the treatment (intercropping) has a significant effect (increase or decrease) compared to the control (monocropping). Conversely, if the 95% CI for the overall effect size of the indicator cross the zero point on the x-axis, we consider the treatment (intercropping) has a insignificant effect compared to the control (monocropping). For studies that reported only the mean value without reporting the standard deviation, we calculated the coefficient of variation (CV) within each observation, then calculate the average CV, and finally multiplied the reported mean value by the average CV to estimate the unreported standard deviation (van Groenigen et al., 2011).

For the α-diversity of soil microbial communities, we chose the Shannon index, Chao1 index, and richness index for representation. For the β-diversity and community structure of soil microbial communities, we extracted the results of the ordination analysis from the different two-dimensional ordination plot and converted them into one-dimensional data. The specific methodological details are derived from a description in a meta-analysis (Zhou et al., 2020). The different two-dimensional ordination plots included non-metric multidimensional scaling (NMDS), principal component analysis (PCA), principal correspondence analysis (PCoA), and redundancy analysis (RDA). After obtaining the precise coordinates of the samples on the first two principal axes, we used the “vegan” package in R to calculate the Euclidean distance between different samples. Subsequently, the logarithmic response ratio (lnRRb) of microbial β-diversity and the logarithmic response ratio (lnRRs) of microbial community structure were calculated using the following equations:


$$\text{lnRRb}=\ln\frac{D_{t}}{D_{c}}=\ln D_{t}-\ln D_{c}$$

$$\text{lnRRs}=\ln\frac{D_{t}}{D_{c}+D_{t}}=\ln D_{t}-\ln(D_{c}+D_{t})$$

Among these, *D*_c_, *D*_t_, *D*_b_, and *D*_c_ + *D*_t_ represent the control (monoculture), treatment (intercropping), between control and treatment, and overall *D*_c_ and *D*_t_, respectively. Based on comprehensive testing, there is no significant deviation between different ranking methods, indicating the current algorithm can compare the responses of microbial β-diversity and community structure to SBI across different studies.

To explain the heterogeneity among studies and infer the importance of these factors for intercropping, we investigated whether the overall effect size of intercropping varies depending on factors such as planting methods, management practices, and sampling strategies. Subgroup analyses were conducted accordingly; nitrogen source types were categorized into mineral fertilizers as nitrogen sources, organic fertilizer as the nitrogen source, or a mixture of mineral fertilizer and organic fertilizer; nitrogen application methods were categorized as basal fertilizer or top dressing; crop types were categorized as leguminous crops or non-leguminous crops; intercropping types were categorized as soybean-maize intercropping or non-soybean-maize intercropping; experimental conditions were categorized as pot experiments or field experiments; sampling areas were categorized as rhizosphere or bulk soil; and microbial types were categorized as bacteria, fungi, and special fungi (such as additive manufacturing frontiers, ammonia-oxidizing bacteria, archaea, comammox, diazotroph, nitrite-oxidizing bacteria, rhizobia).

### 2.3 Publication bias and statistical analysis

The fail-safe method was used to assess publication bias in the current meta-analysis (Rosenthal, 1986). If the fail-safe number is greater than 5*n* + 10, the results are robust and there is no publication bias. The results showed that the fail-safe number for all variables except soybean yield was greater than 5*n* + 10. For soybean yield, we further used Kendall’s tau rank test to assess publication bias, and the results indicated that the *p*-value was greater than 0.05, indicating no publication bias (Kendall, 1938). Therefore, all response ratio metrics included in the analysis showed no publication bias (Supplementary Table S1).

We used a comprehensive test (Q_M_) to compare the responses of various microbial traits to SBI under different categories of regulatory factors. If the Q_M_ test indicated a regulatory factor had a significant (*p* < 0.05) effect, we used the glht () function in the “multcomp” software package to perform Tukey’s HSD test as a *post hoc* comparison to assess the differences between different category levels.

In order to investigate the relationship between microbial community traits and soil environmental factors, we used the package “linkET” for Mantle analysis, and for important environmental factors, we used the “bestFitM” function in the package “BestFitM” to select the appropriate model for regression analysis based on the Akaike information criterion and Bayesian information criterion (Oksanen et al., 2013). Model selection analysis was performed using the random forest method in SPSS Pro to explore the relative importance of influencing factors, including annual precipitation, annual temperature, experimental period, RDT, pRDT, pHi, SOCi, TNi, NFR (nitrogen application rate), soybean rows, and spacing between soybeans and other crops. All visualizations were created in “Origin 2024.”

## 3 Results

### 3.1 Overall impact of soybean-based intercropping on soil microbial communities

**Figure 1:**
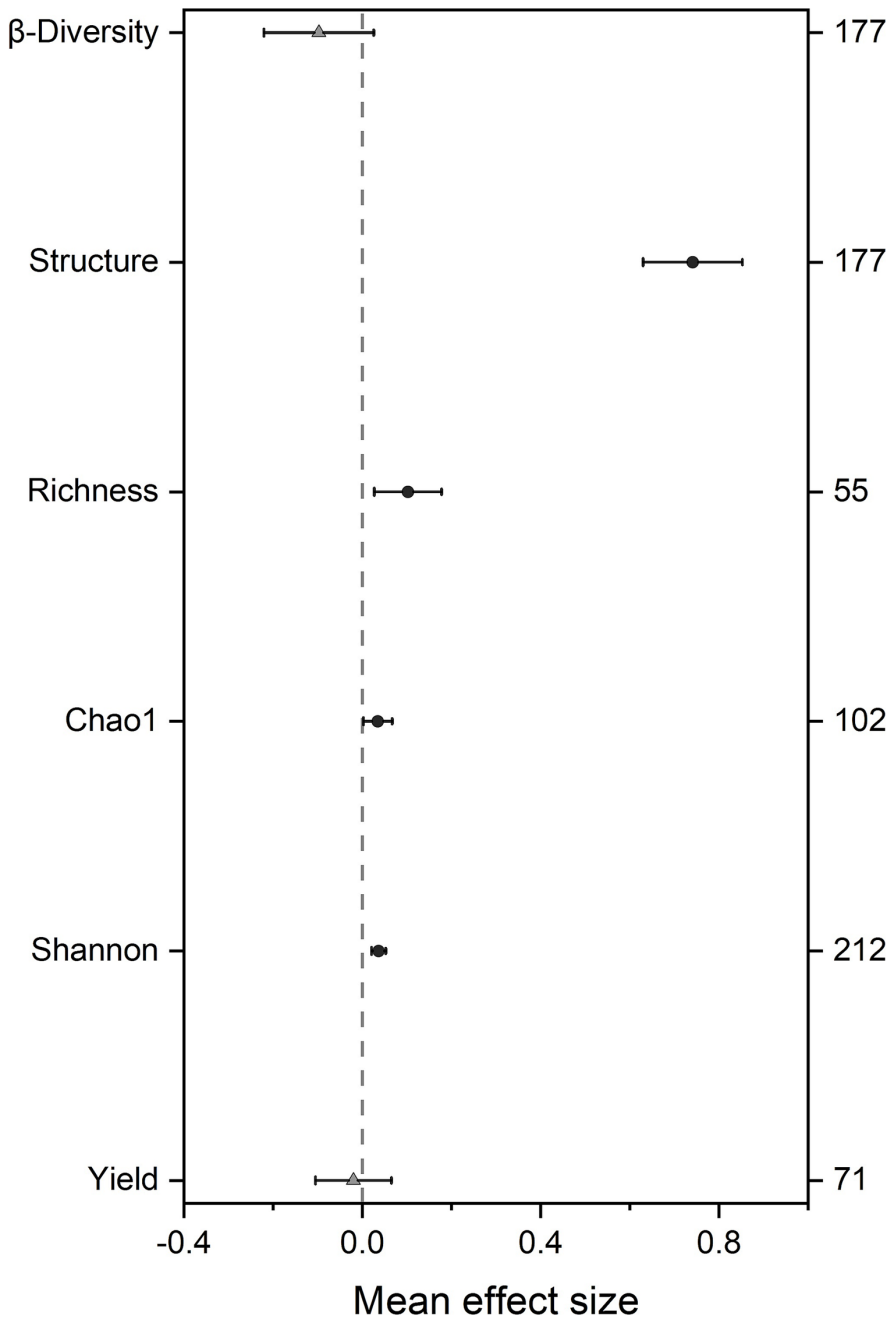
Effect of soybean-based intercropping (SBI) on the soil microbial community parameters and yield. Mean bar values are expressed as the mean effect size of each variable with 95% confidence intervals (CIs). Shannon diversity index (Shannon), Richness index (Richness), Chao 1 index (Chao 1) were categorized as microbial community α-diversity parameters, and structure, β-diversity were categorized as microbial community structure and β-diversity parameters, respectively. Circle indicates 95% CIs for the overall effect size of the indicator does not cross the zero point; Triangle indicates 95% CIs for the overall effect size of the indicator cross the zero point on the x-axis.

Overall, SBI significantly altered soil microbial community structure (effect size = 0.741, 95% CI: 0.629–0.852) and α-diversity indices, including richness (effect size = 0.102, 95% CI: 0.026–0.178), Chao1 (effect size = 0.034, 95% CI: 0.002–0.067), and Shannon (effect size = 0.036, 95% CI: 0.020–0.053), all increased, while there was no significant effect on soil microbial community β-diversity (effect size = −0.098, 95% CI: −0.221 to 0.026). We found soybean yield showed the smallest response to intercropping patterns (effect size = −0.020, 95% CI: −0.105 to 0.065) (Figure 1). Additionally, different detection methods had no significant effect on soil microbial β-diversity, while the interpretation of structure using PCA may introduce some bias. Overall, the use of different detection methods in different studies had little impact on experimental results (Supplementary Figure S2).

**Figure 2:**
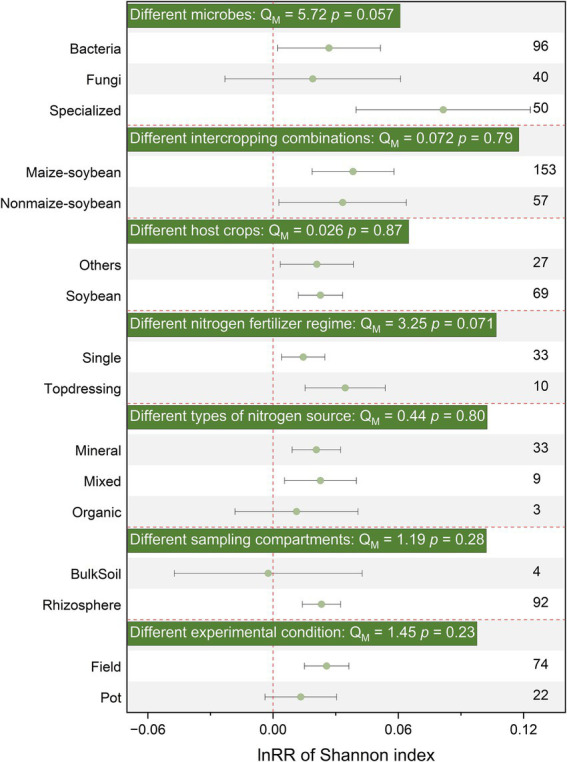
Effects of SBI on soil microbial community Shannon parameters among different microbes, different intercropping combinations, different host crops strip, different nitrogen fertilizer regime, different types of nitrogen source, different sampling sites, different experimental condition. The vertical dashed line was drawn at mean response ratio (RR) = 0. Error bars represent 95% confidence intervals (CIs), and the number on the y-axis indicate the number of observations. If 95% CI does not overlap the zero line, the effect of warming on a variable is considered significant. If the 95% CI overlaps the zero line, the effect of warming is considered insignificant. A *p* < 0.05 indicates a significant difference between the subgroups. The significances of various moderators are tested by omnibus test (Q_M_).

**Figure 3:**
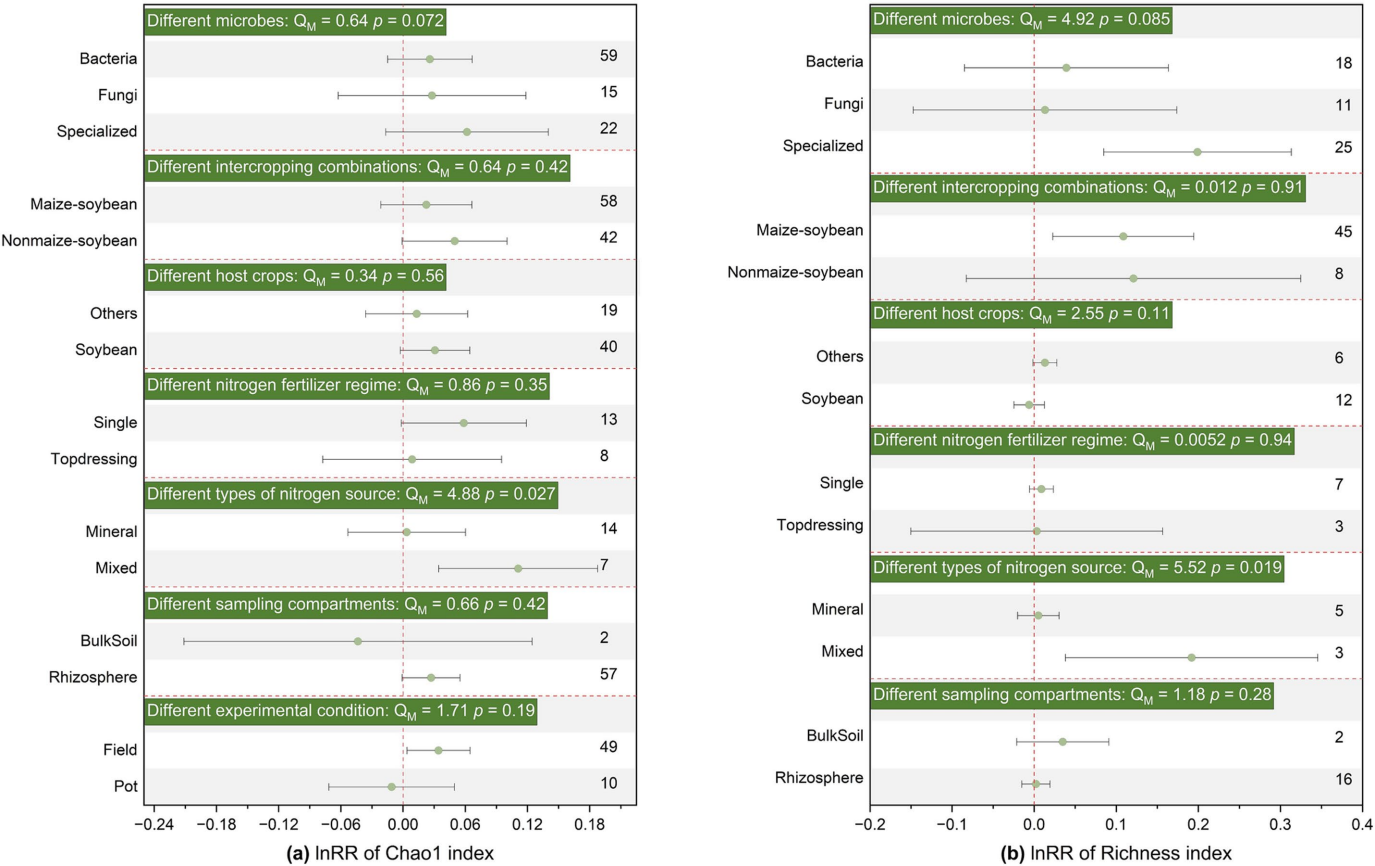
Effects of SBI on soil microbial community Chao1 **(a)** and Richness **(b)** parameters among different microbes, different intercropping combinations, different host crops strip, different nitrogen fertilizer regime, different types of nitrogen source, different sampling sites, different experimental conditions. The vertical dashed line was drawn at mean response ratio (RR) = 0. Error bars represent 95% confidence intervals (CIs), and the number on the y-axis indicate the number of observations. If 95% CI does not overlap the zero line, the effect of warming on a variable is considered significant. If the 95% CI overlaps the zero line, the effect of warming is considered insignificant. A *p* < 0.05 indicates a significant difference between the subgroups. The significances of various moderators are tested by omnibus test (Q_M_).

Additionally, SBI generally did not significantly affect Shannon, Chao1, richness, β-diversity, or structure for different soil microbial groups, without distinguishing between bacteria, fungi, and functional microorganisms (Figures 2, 3; Supplementary Figure S3). Therefore, subsequent analyses were conducted on the entire microbial community without distinguishing specific groups.

### 3.2 Effects of management and sampling factors on rhizosphere microbial community traits

The application of basal fertilizer had a positive effect on Shannon index (effect size = 0.014, 95% CI: 0.004–0.025) and changed community structure (effect size = 0.514, 95% CI: 0.202–0.826), but had no significant effect on α-diversity or β-diversity (Figures 2, 3; Supplementary Figure S3). Notably, the response of microbial α-diversity can be affected by nitrogen fertilizer type. In the SBI, the application of mixed fertilizer has a positive effect on chao1 and richness (Figure 3) Both inorganic (effect size = 0.276, 95% CI: 0.101–0.452) and organic nitrogen (effect size = 0.967, 95% CI: 0.441–1.492) fertilizer application changed microbial community structure, while the effect was more prolonged under organic nitrogen fertilizer than that of inorganic nitrogen fertilizer (*p* < 0.05, Supplementary Figure S3). Soil microbial Shannon index and community structure was significantly enhanced (effect size = 0.023, 95% CI: 0.012–0.033) or changed (effect size = 0.568, 95% CI: 0.372–0.763) by SBI in the in soybean strip, respectively. Sampling in non-soybean strip showed positive responses to soil microbial Shannon index (effect size = 0.021, 95% CI: 0.003–0.039) and changed community structure (effect size = 0.690, 95% CI: 0.425–0.954) (Figure 2; Supplementary Figure S3). However, sampling sites had no significant effect on chao1, richness, and β-diversity (Figure 3; Supplementary Figure S3). Additionally, sampling sites in rhizosphere significantly affected the Shannon index and community structure, increasing or changing them by (effect size = 0.023, 95% CI: 0.014–0.032) and (effect size = 0.622, 95% CI: 0.464–0.780), respectively; conversely, bulk soil did not significantly affect Shannon diversity or structure (Figure 2; Supplementary Figure S3).

**Figure 4:**
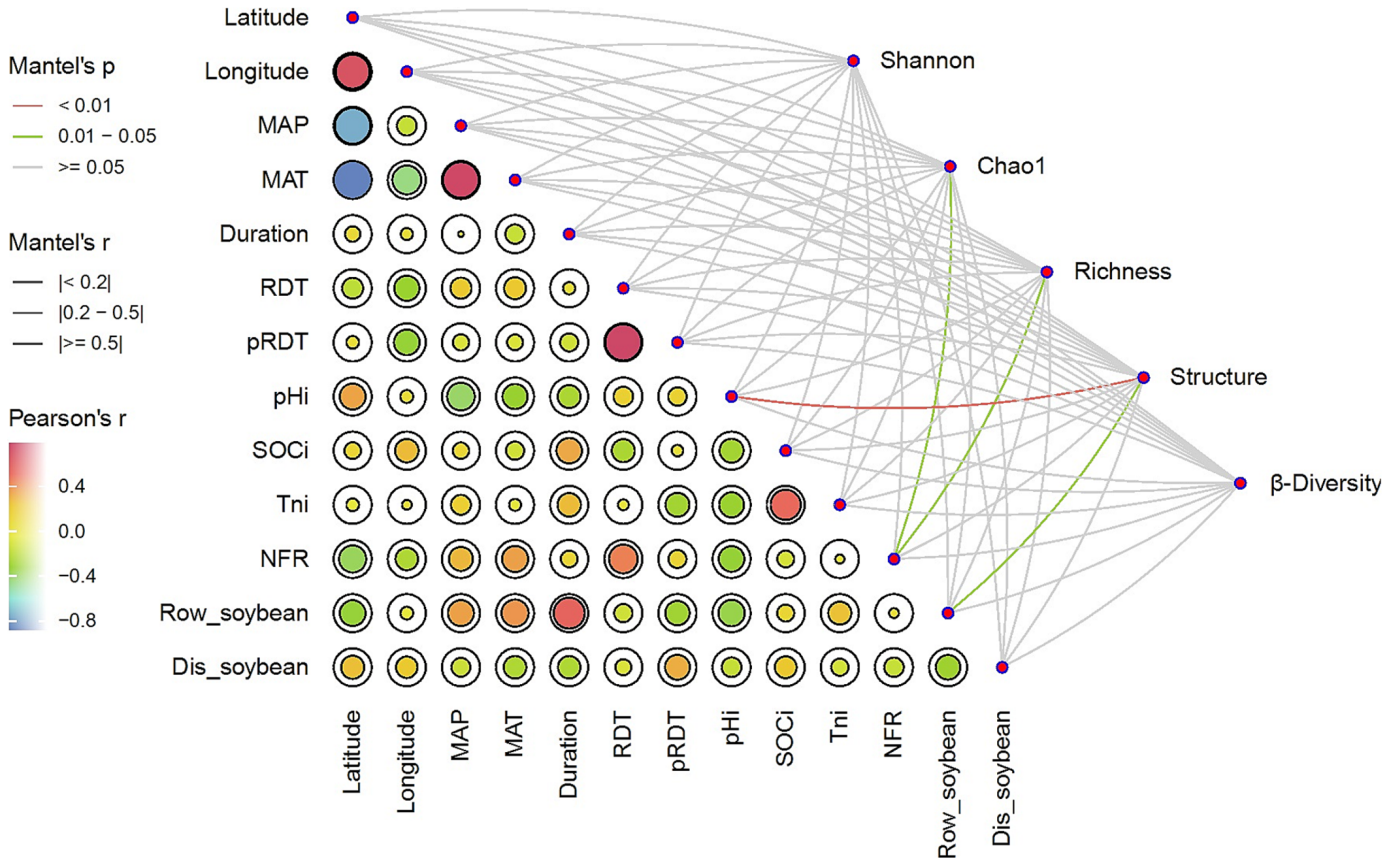
Pairwise comparisons of environmental factors are shown, with a color gradient denoting Pearson correlation coefficients. Soil microbial community parameters was related to each environmental factor by partial Mantel tests. Edge width corresponds to the Mantel’s *r* statistic for the corresponding distance correlations, and edge color denotes the statistical significance based on 9,999 permutations.

**Figure 5:**
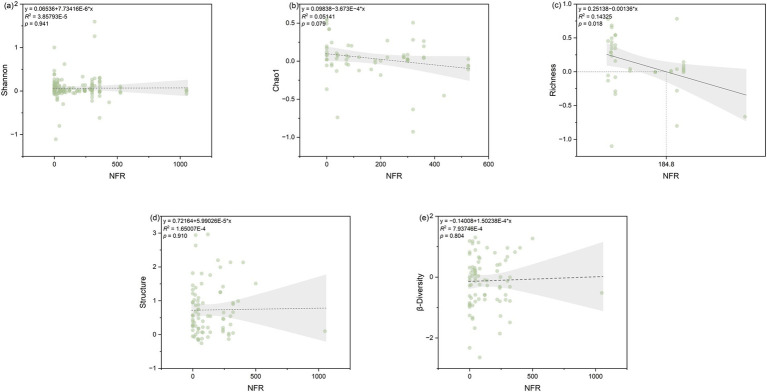
Relationship between the response ratio of soil microbial community index, namely **(a)** Shannon, **(b)** Chao1, **(c)** richness, **(d)** structure, and **(e)** β-diversity to the NFR (nitrogen application rate) as affected by SBI. The grey zone indicates 95% confidence intervals. The *R*^2^ and *p*-value of the models are noted.

NFR was significantly (*p* < 0.05) correlated with both Chao1 and richness indices (*p* < 0.05), the number of soybean rows (Row-soybean) was significantly (*p* < 0.05) correlated with soil microbial structure, Dis-soybean had no significant effect on Shannon, RDT had no significant effect on Shannon, and pRDT had no significant effect on Shannon (Figure 4). Further regression analysis revealed that NFR was significantly correlated with richness (Figure 5).

### 3.3 Effects of soil and environmental factors on soil microbial community structure

Our meta-analysis revealed significant differential effects of environmental factors on soil microbial communities in SBI systems. Soil pHi had a highly significant effect on soil microbial community structure (*p* < 0.05). In contrast, MAP, MAT and SOCi had no significant effect on soil microbial community structure and soil microbial diversity (Figure 4).

### 3.4 Key predictors of soil microbial community traits

Random forest analysis was employed to identify key factors driving changes in microbial community diversity and structure. The results revealed that NFR was the most important factor in predicting Chao1, richness, and Shannon indices. Moreover, we found that taxa were the most influential factor affecting β-diversity, while sampling sites had the strongest impact on microbial community structure (Supplementary Figure S4).

### 3.5 Relationship between soil microbial diversity and soybean yield

**Figure 6:**
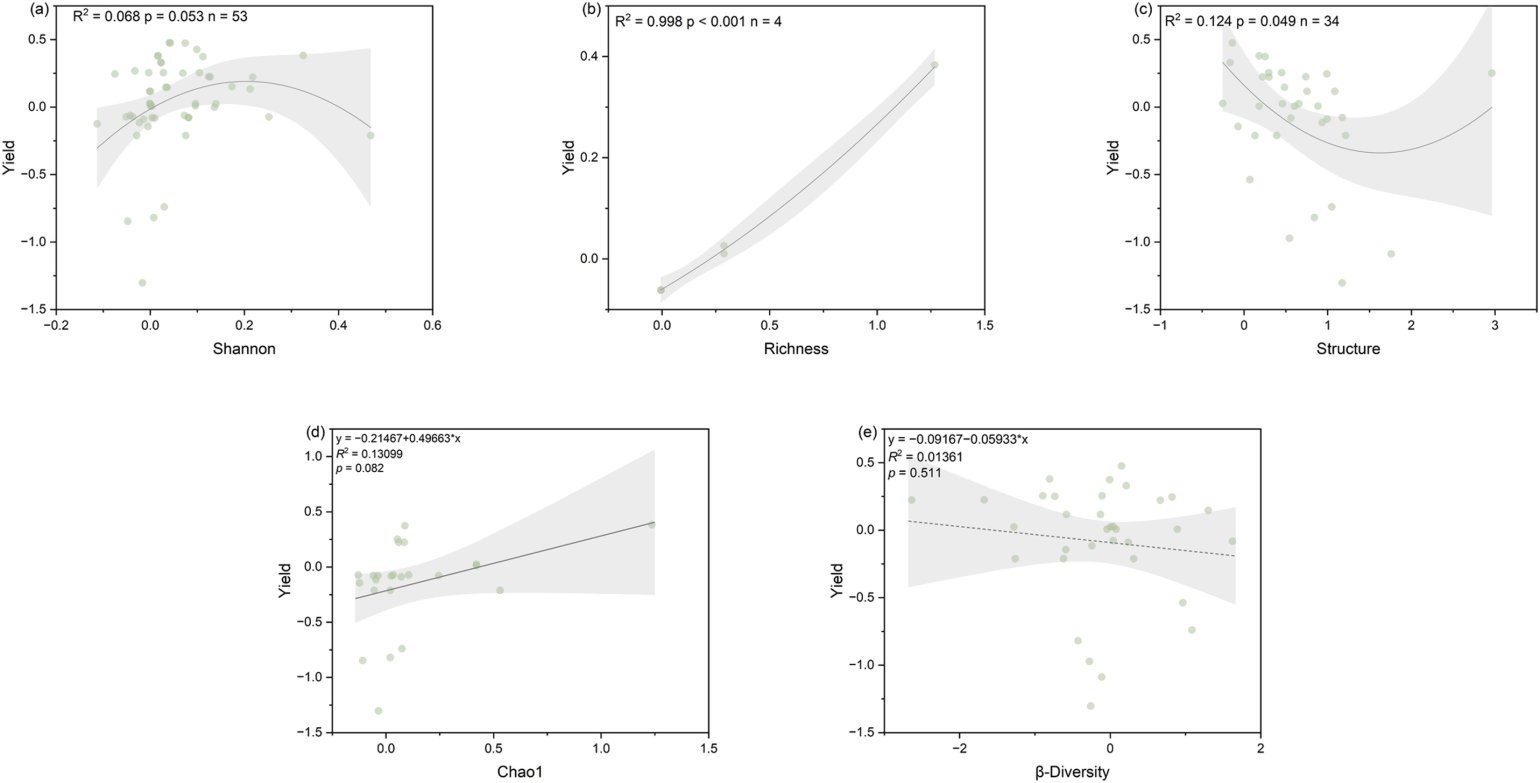
Relationship between the response ratio of soil microbial community index, namely **(a)** Shannon, **(b)** Chao1, **(c)** richness, **(d)** structure, and **(e)** β-diversity to the soybean yield as affected by SBI. The grey zone indicates 95% confidence intervals. The *R*^2^ and *p*-value of the models are noted.

Chao1 (*R*^2^ = 0.131, *p* = 0.082) showed a marginally significant positive correlation with soybean yield, while richness (*R*^2^ = 0.998, *p* < 0.001) showed a significant positive correlation with soybean yield. Shannon diversity exhibited a parabolic relationship with soybean yield, and within the range of 0.008–0.401, the lnRR value of soybean yield was greater than 0, and the response ratio of soybean yield is maximum when Shannon was 0.212, showing the most significant effect on soybean yield increase under intercropping (Figure 6). Environmental and soil factors also influenced yield. MAT, MAP, SOCi, and TNi are positively correlated with yield, while NFR is not significantly correlated with yield (Supplementary Figure S5). Additionally, pHi is negatively correlated with community structure (Supplementary Figure S6).

## 4 Discussion

### 4.1 Soybean-based intercropping significantly increases soil microbial α-diversity and alters community structure

We found that SBI significantly altered the structure and increased the α-diversity (Shannon index, Chao1 index, and species richness) of soil microbial communities, suggesting that these shifts were driven by SBI-induced alterations in soil nutrients (Zhang et al., 2023). The primary reason is that intercropping particularly leads to resource complementarity effects and ecological niche differentiation between soybeans and their intercropping partners (Li et al., 2014; Ren et al., 2014), increasing root exudate diversity. On one hand, this provides more substrates required by microorganisms, directly enhancing microbial diversity. For example, soybean-maize intercropping increases the secretion of sugars, amino acids, organic acids, and lipids in root exudates. This enhanced secretion provides a rich carbon source supply for rhizosphere microorganisms, thereby promoting the improvement of rhizosphere microbial diversity (Zhang S. et al., 2024). On the other hand, under intercropping, the substances secreted by the root systems of soybeans and their companion crops participate in biogeochemical reactions in the soil environment, enhancing the availability of nutrients such as available nitrogen and available phosphorus, thereby improving the microbial colonization environment and indirectly increasing microbial diversity (Li et al., 2014; Withers et al., 2020).

Specifically, under the SBI system, the Shannon index showed significant increases in multiple subgroups, while Chao1 and richness remained largely unchanged. This indicates under soybean intercropping, the Shannon index is more sensitive to changes in microbial species with higher abundance, as it simultaneously considers both richness and evenness; while Chao1 is to the proxy for changes in rare microbial taxa (Wang et al., 2025). Rare groups remain stable under multiple environmental factor changes, while abundant groups exhibit more sensitive changes (Liang et al., 2020). Therefore, intense nutrient and physical environmental changes may regulate microbial community evenness by influencing the relative abundance of abundant taxa, while having a smaller impact on the rare ones. As a result, the response patterns of Shannon and Chao1 differ. Additionally, the functional gene expression levels of abundant taxa were higher than those of rare taxa. This further indicates that SBI promotes the environmental adaptability of abundant taxa These microorganisms can efficiently carry out functions such as C, N, S, and P cycling, thereby driving biogeochemical cycles (Zhu et al., 2023).

However, SBI had minor effect on soil microbial β-diversity, partially refuting our first hypothesis. β-diversity focuses on comparing biodiversity between samples, specifically comparing the microbial community composition of different samples (Gou et al., 2019). This may because that soil microbial β-diversity is more influenced by changes in aboveground plant biomass than simply by plant species composition (Li et al., 2018). SBI significantly altering soil microbial community structure, which suggests that intercropping not only increases species α-diversity but also exerts effects by altering microbial community composition. This can be explained by the synergistic interactions between soybeans and companion crops, which alter soil microenvironments and resource availability, and promoting the growth of specific microbial taxa (Zhang et al., 2023). The interaction between soil microbial community diversity or composition and other factors leads to changes in soil microenvironments, resulting in differences in microbial community diversity and composition under SBI (Sun et al., 2020; Zhao et al., 2024).

### 4.2 Key factors influencing soil microbial response to soybean-based intercropping

#### 4.2.1 Response of soil microorganisms to soybean-based intercropping under different nitrogen fertilizer conditions

Different fertilization types influence soil microbial community structure and diversity (Tang et al., 2017). Through random forest modeling, it was found under SBI, NFR was the most significant factor influencing chao1, richness, and Shannon diversity, supporting the second hypothesis. Moreover, NFR had a positive effect on chao1 and richness in the SBI system (0.01 < Mantel’s *p* < 0.05), with NFR increases, Chao1 and richness decrease. This aligns with findings from studies on microbial communities in dryland red soils, where the chao1 index decreased by 46.7% under nitrogen application compared to no fertilization (Liu et al., 2020). Moderate nitrogen fertilization increases microbial Shannon index, but excessive nitrogen fertilization (when NFR exceeds 184.8 kg N ha^−1^) reduces it (Figure 5c). This can be explained by the fact after excessive nitrogen fertilization, the general plant utilization rate of fertilizers is only 30–50%, with the remainder converted into nitrate through nitrification in the soil. This leads to soil acidification and neutralization reactions with inorganic carbon in the soil, resulting in the loss of soil carbon (Bai et al., 2023). Ultimately, this leads to complete soil degradation, which inevitably has an adverse effect on bacterial community diversity in some way (Zhang L. et al., 2024). Our research showed under SBI, soil microbial diversity decreases when NFR exceeds 184.8 kg N ha^−1^. Previous studies have indicated 180 kg N ha^−1^ is an appropriate nitrogen application rate for intercropping, which most effectively promotes soybean nodulation and maize nitrogen accumulation. While exceeding this threshold to 240 kg N ha^−1^ constitutes excessive nitrogen application (Yong et al., 2018; Li et al., 2022). This is consistent with the results of our meta-analysis. Previous studies have found that in maize-soybean intercropping, nutrient competition is more important than above-ground light competition, and underground benefits contribute to above-ground growth, thereby increasing soybean yield (Chen et al., 2017). Therefore, based on the results of our meta-analysis, under SBI systems, nitrogen application influences nodulation and crop biomass accumulation by regulating microbial community diversity, thereby affecting soybean yield under intercropping conditions. We found nitrogen application rates around 180 kg N ha^−1^ are most beneficial. Under SBI, conventional nitrogen application is 240 kg N ha^−1^; however, our research indicates reduced nitrogen application is more beneficial for enhancing soybean and companion crop yields and dry matter accumulation. Therefore, SBI with reduced nitrogen input provides a highly useful method for improving land productivity and avoiding environmental pollution.

Under a SBI system, the effect of nitrogen fertilizer type on soil microbial α-diversity is closely related to fertilizer type, with organic nitrogen fertilizers having a significantly greater effect on altering soil microbial community structure than inorganic nitrogen fertilizers. Changes in microbial community structure are primarily due to differences in total soil carbon and nitrogen content (Yu et al., 2013). Organic fertilizers increased soil fertility more than mineral fertilizers, thereby modifying the microbial community structure in intercropped soils (Dang et al., 2022). Mineral fertilizers, however, reduce microbial diversity, including beneficial microbial groups for plants (Francioli et al., 2016), which aligns with our findings. Additionally, mixed fertilizers have a positive impact on the Chao1 index and richness, consistent with the findings of (Wang et al., 2017). Under SBI, organic fertilizer treatments exhibited higher soil microbial diversity indices than inorganic fertilizer treatments. After applying organic fertilizer, the soil richness index and Shannon index significantly increased compared to the control group. Organic nitrogen fertilizers can provide soybeans and their companion crops with more sustained nutrient release and more complex microbial substrates than inorganic nitrogen fertilizers (Geisseler and Scow, 2014). Furthermore, soil microbial diversity is the result of the combined effects of multiple soil environmental factors. In SBI systems, the application of organic fertilizers alters the microbial growth environment, thereby significantly influencing soil microbial diversity (Huang et al., 2023). Organic fertilizers not only provide abundant substrates for soybeans and their companion crops but also improve the physical and chemical properties of soil under intercropping, optimize the ecological structure of soil microorganisms, maintain the ecological environment of soil microorganisms, and increase the diversity of microbial community. The proliferation of microorganisms further accelerates the degradation and transformation of organic matter in soil, thereby enhancing soil fertility and creating a virtuous cycle (Kong et al., 2022).

#### 4.2.2 Response of soil microorganisms to SBI under other important factors

Previous studies have found microorganisms showing promise in laboratory settings may lack key traits necessary for widespread adoption in sustainable and productive agricultural field systems (Parnell et al., 2016). Our meta-analysis revealed that SBI in field experiments significantly increased soil microbial Shannon and Chao1 indices, whereas the indices in pot experiments remain unchanged. This suggests SBI was more likely to enhance microbial diversity, thereby effectively reflecting key microbial traits in the field environment. Compared to pot experiments, field experiments have lower soil bulk density, providing ample space for root interactions, resulting in strong root interactions between soybeans and their companion crops (Li et al., 2002). This root niche interaction is crucial for field efficacy, and thus organisms with greater multifunctionality will exhibit higher efficacy under various field conditions (Parnell et al., 2016). Surprisingly, the row configuration of intercropping had minimal linkage with microbial community traits, indicating the second hypothesis is not fully supported. This suggests the consistent benefits of SBI to soil microbial community diversity, regardless the variations in row configurations. Furthermore, the changes in microbial community traits were decoupled with soybean density. The stability of microbial communities under different intercropping densities may be attributed to functional redundancy, where multiple groups perform similar ecological functions (Jurburg and Salles, 2015). Functional redundancy refers to the phenomenon where the more organisms involved in a specific process, the less likely that process will be affected if some organisms lose their ability or are removed (Andrén et al., 1999). Therefore, functional redundancy obscures the connection between microbial taxonomy and functional traits (Schimel, 1995), particularly when studying more broadly defined processes (e.g., carbon cycling of rhizosphere root exudates), where many taxonomic groups may be responsible for the same biogeochemical function (Hungria et al., 2009).

### 4.3 Relationship between rhizosphere microbial community traits and soybean yield

A meta-analysis study found that compared to monoculture, intercropping significantly reduced soybean yield (Yang et al., 2024). However, according to our meta-analysis, when focusing on the changes of soil microbial community traits, soybean yield did not decrease under intercropping. This suggests changes in microbial community structure and increases in the Chao1 and Shannon indices are beneficial for narrow the yield gap between intercropping and monoculture. These can be explained by the improved soil environment under intercropping, which lead to interspecific competition complementarity and niche differentiation, thereby influencing crop photosynthesis, root growth and development, and root architecture distribution (Brooker et al., 2015; Bukovsky-Reyes et al., 2019). Moreover, intercropping also alters the root-zone environment, and influencing the colonization and flourish of rhizosphere microorganisms, increasing their chao1 and Shannon indices. Crop roots influence above-ground plant development, ultimately affecting yield and preventing yield reduction (Li, 2016).

Our meta-analysis demonstrates that NFR exerts a positive yet indirect effect on soybean yield in intercropping systems. The observed correlations reveal that NFR shows a significant positive relationship with soil microbial α-diversity metrics (Chao1 and species richness), while these diversity indices themselves exhibit marginal yet biologically meaningful associations with yield. This suggests that NFR enhances intercropped soybean yield primarily through its stimulation of soil microbial diversity. Notably, the Shannon diversity index displayed a unimodal relationship with yield, where yield initially increased but declined beyond optimal diversity levels. This nonlinear response supports our third hypothesis that strategically enhancing microbial diversity can promote yield formation in intercropped soybeans, though excessive diversity may prove counterproductive. These findings align with existing literature proposing that microbial community shifts can improve crop nitrogen use efficiency, potentially explaining the observed yield benefit (Dang et al., 2020). However, when the Shannon index becomes too high, yield decreases, indicating overly complex and homogeneous microbial community structures may not necessarily benefit soybean yield. This may be related to an increase in harmful microorganisms, and additionally, overly complex microbial communities may lead to competition between microorganisms and crop roots for nutrients. Studies have shown reductions in soil biodiversity and basic soil functions (such as nutrient cycling) can limit plant growth and crop yields (Stefan et al., 2021), while increases in chao1 do not lead to yield declines, suggesting rare microorganisms play a more significant role, consistent with previous research (Mendes et al., 2014; Xiao et al., 2022). Studies have shown fungal communities associated with crops are dominated by a few abundant taxonomic groups primarily belonging to Sordariomycetes and Dothideomycetes, while most of the diversity in fungal communities is represented by rare taxonomic groups. Rare taxa play a crucial role in fungal co-occurrence networks and ecosystem functions, such as crop yield and soil enzyme activity. Rare taxa are essential for maintaining crop microbial community stability and ecosystem functions (Xiong et al., 2021).

## 5 Conclusion

In this study, a global database was established to investigate the effects of SBI on soil microbial diversity, richness, and community structure, followed by a meta-analysis at the global scale. The results indicated SBI significantly influences soil microbial community metrics. SBI systems enhance soil microbial α-diversity and alter community structure. A random forest model identified NFR as the primary factor influencing chao1, richness, and Shannon diversity. Specifically, nitrogen fertilization regimes play a key role in soil microbial responses to SBI, with organic fertilizer treatments exhibiting higher soil microbial diversity indices than inorganic fertilizer treatments under SBI. Furthermore, the soil microbial Chao1 index exhibits a linear positive correlation with yield, while the Shannon index shows a quadratic relationship with yield, rather than soil microbial β-diversity, indicating that regulating changes in alpha diversity, which characterizes species abundance, is key to enhancing crop yield. In conclusion, these findings reveal the response of soil microorganisms to SBI, highlighting the potential for promoting soil health and agricultural sustainability through the regulation of microbial communities.

## Data Availability

The original contributions presented in the study are included in the article/Supplementary material, further inquiries can be directed to the corresponding author.
