# Supplementary material for: Response of soil microbial community diversity and structure to soybean-based intercropping and its effects on yield
Source: Front Microbiol. 2025 Aug 29;16:1658783. doi: 10.3389/fmicb.2025.1658783 (PMC12425927; doi:10.3389/fmicb.2025.1658783)
Supplement: Supplementary file 1 [file Data_Sheet_1.docx]

Supplementary Material

**Supplementary Data**

Text S1 Data sources of this meta-analysis study (studies in Chinese but with English abstract are marked in blue):

1. Solanki, M. K., Wang, Z., Wang, F. Y., Li, C. N., Gupta, C. L., Singh, R. K., ... & Li, Y. R. (2020). Assessment of diazotrophic proteobacteria in sugarcane rhizosphere when intercropped with legumes (peanut and soybean) in the field. Frontiers in microbiology, 11, 1814.
2. Wang Xiaohui (2023). Effects of Rhizoctonia solani on Plant and Soil Factors in Solanum nigrum/Soybean Intercropping System under Cd Stress (Doctoral Dissertation, Heilongjiang University) Dr https://link.cnki.net/doi/10.27123/d.cnki.ghlju.2023.000018 doi:10.27123/d.cnki.ghlju.2023.000018.
3. Cheng, Z., Meng, L., Yin, T., Li, Y., Zhang, Y., & Li, S. (2023). Changes in soil rhizobia diversity and their effects on the symbiotic efficiency of soybean intercropped with maize. Agronomy, 13(4), 997.
4. Yang, S. D., Xiao, J., Huang, Z. Y., Qin, R. L., He, W. Z., Liu, L. M., ... & Tan, H. W. (2021). Comparison of soil biological properties and bacterial diversity in sugarcane, soybean, mung bean and peanut intercropping systems. J Agr Sci, 13(8), 54-68.
5. Chen, D., Wang, C., Ma, X., Chen, K., Wang, Z., Wang, Q., ... & Shen, W. (2023). Dynamic changes in soil fungal communities and functional groups in response to sugarcane/soybean intercropping with reduced nitrogen fertilizer application. Biology and Fertility of Soils, 59(4), 363-378.
6. Li, H., Luo, L., Tang, B., Guo, H., Cao, Z., Zeng, Q., ... & Chen, Z. (2020). Dynamic changes in the rhizosphere bacterial community in monoculture and intercropping maize and soybean during various crop growth stages.
7. Li, H., Luo, L., Tang, B., Guo, H., Cao, Z., Zeng, Q., ... & Chen, Z. (2022). Dynamic changes of rhizosphere soil bacterial community and nutrients in cadmium polluted soils with soybean-corn intercropping. BMC microbiology, 22(1), 57.
8. Lu, X. (2022). Effect of intercropping soybean on the diversity of the rhizosphere soil arbuscular mycorrhizal fungi communities in wheat fields. CLEAN–Soil, Air, Water, 50(6), 2100014.
9. Liu, Y., Yang, H., Liu, Q., Zhao, X., Xie, S., Wang, Z., ... & Chen, B. (2021). Effect of two different sugarcane cultivars on rhizosphere bacterial communities of sugarcane and soybean upon intercropping. Frontiers in microbiology, 11, 596472.
10. Li, L., Zou, Y., Wang, Y., Chen, F., & Xing, G. (2022). Effects of corn intercropping with soybean/peanut/millet on the biomass and yield of corn under fertilizer reduction. Agriculture, 12(2), 151.
11. Herrmann, L., Chotte, J. L., Thuita, M., & Lesueur, D. (2014). Effects of cropping systems, maize residues application and N fertilization on promiscuous soybean yields and diversity of native rhizobia in Central Kenya. Pedobiologia, 57(2), 75-85.
12. Li, Z. H. O. U., Ping, C. H. E. N., Qing, D. U., Ting, P. A. N. G., Chun, S. O. N. G., Xiao-chun, W. A. N. G., ... & Tai-wen, Y. O. N. G. (2019). Effects of maize-soybean relay intercropping on crop nutrient uptake and soil bacterial community. Journal of Integrative Agriculture, 18(9), 2006-2018.
13. Liu, Y., Ma, W., He, H., Wang, Z., & Cao, Y. (2021). Effects of sugarcane and soybean intercropping on the nitrogen-fixing bacterial community in the rhizosphere. Frontiers in Microbiology, 12, 713349.
14. Wei, B., Zhang, J., Wen, R., Chen, T., Xia, N., Liu, Y., & Wang, Z. (2021). Genetically modified sugarcane intercropping soybean impact on rhizosphere bacterial communities and co-occurrence patterns. Frontiers in Microbiology, 12, 742341.
15. Lian, T., Mu, Y., Jin, J., Ma, Q., Cheng, Y., Cai, Z., & Nian, H. (2019). Impact of intercropping on the coupling between soil microbial community structure, activity, and nutrient-use efficiencies. PeerJ, 7, e6412.
16. Xiao, X., Han, L., Chen, H., Wang, J., Zhang, Y., & Hu, A. (2023). Intercropping enhances microbial community diversity and ecosystem functioning in maize fields. Frontiers in Microbiology, 13, 1084452.
17. ZHONG, Y., LIANG, L., XU, R., XU, H., SUN, L., & LIAO, H. (2022). Intercropping tea plantations with soybean and rapeseed enhances nitrogen fixation through shifts in soil microbial communities. Frontiers of Agricultural Science and Engineering, 9(3), 344-355.
18. Zhang, G., Yang, H., Zhang, W., Bezemer, T. M., Liang, W., Li, Q., & Li, L. (2023). Interspecific interactions between crops influence soil functional groups and networks in a maize/soybean intercropping system. Agriculture, Ecosystems & Environment, 355, 108595.
19. You, Y., Wang, L., Liu, X., Wang, X., Jiang, L., Ding, C., ... & Zhao, X. (2024). Interspecific plant interaction structures the microbiomes of poplar-soil interface to alter nutrient cycling and utilization. Microbiology Spectrum, 12(2), e03368-23.
20. Zhang, S., Meng, L., Hou, J., Liu, X., Ogundeji, A. O., Cheng, Z., ... & Li, S. (2022). Maize/soybean intercropping improves stability of soil aggregates driven by arbuscular mycorrhizal fungi in a black soil of northeast China. Plant and Soil, 481(1), 63-82.
21. Liu, J., Li, Y., Han, C., Yang, D., Yang, J., Cade-Menun, B. J., ... & Sui, P. (2022). Maize-soybean intercropping facilitates chemical and microbial transformations of phosphorus fractions in a calcareous soil. Frontiers in Microbiology, 13, 1028969.
22. Liu, M., & Zhao, H. (2023). Maize-soybean intercropping improved maize growth traits by increasing soil nutrients and reducing plant pathogen abundance. Frontiers in Microbiology, 14, 1290825.
23. Lu, M., Zhao, J., Lu, Z., Li, M., Yang, J., Fullen, M., ... & Fan, M. (2025). Maize–soybean intercropping increases soil nutrient availability and aggregate stability. Plant and Soil, 506(1), 441-456.
24. Chang, X., Wei, D., Zeng, Y., Zhao, X., Hu, Y., Wu, X., ... & Yang, W. (2022). Maize-soybean relay strip intercropping reshapes the rhizosphere bacterial community and recruits beneficial bacteria to suppress Fusarium root rot of soybean. Frontiers in Microbiology, 13, 1009689.
25. Chifflot, V., Rivest, D., Olivier, A., Cogliastro, A., & Khasa, D. (2009). Molecular analysis of arbuscular mycorrhizal community structure and spores distribution in tree-based intercropping and forest systems. Agriculture, Ecosystems & Environment, 131(1-2), 32-39.
26. Zeng, H., Yu, L., Liu, P., Wang, Z., Chen, Y., & Wang, J. (2021). Nitrogen fertilization has a stronger influence than cropping pattern on AMF community in maize/soybean strip intercropping systems. Applied Soil Ecology, 167, 104034.
27. Yu, L., Tang, Y., Wang, Z., Gou, Y., & Wang, J. (2019). Nitrogen-cycling genes and rhizosphere microbial community with reduced nitrogen application in maize/soybean strip intercropping. Nutrient Cycling in Agroecosystems, 113, 35-49.
28. Zhang, R., Mu, Y., Li, X., Li, S., Sang, P., Wang, X., ... & Xu, N. (2020). Response of the arbuscular mycorrhizal fungi diversity and community in maize and soybean rhizosphere soil and roots to intercropping systems with different nitrogen application rates. Science of the Total Environment, 740, 139810.
29. Shao, M., Wang, C., Zhou, L., Peng, F., Zhang, G., Gao, J., ... & Zhao, Q. (2023). Rhizosphere soil properties of waxy sorghum under different row ratio configurations in waxy sorghum-soybean intercropping systems. Plos one, 18(7), e0288076.
30. Solanki, M. K., Wang, F. Y., Wang, Z., Li, C. N., Lan, T. J., Singh, R. K., ... & Li, Y. R. (2019). Rhizospheric and endospheric diazotrophs mediated soil fertility intensification in sugarcane-legume intercropping systems. Journal of Soils and Sediments, 19, 1911-1927.
31. Zhang, T., Yu, L., Shao, Y., & Wang, J. (2023). Root and hyphal interactions influence N transfer by arbuscular mycorrhizal fungi in soybean/maize intercropping systems. Fungal Ecology, 64, 101240.
32. Shen, F. T., & Lin, S. H. (2021). Shifts in bacterial community associated with green manure soybean intercropping and edaphic properties in a tea plantation. Sustainability, 13(20), 11478.
33. Cao, X., Liu, S., Wang, J., Wang, H., Chen, L., Tian, X., ... & Qiao, Z. (2017). Soil bacterial diversity changes in different broomcorn millet intercropping systems. Journal of Basic Microbiology, 57(12), 989-997.
34. Yang, Z., Zhu, Q., Zhang, Y., Jiang, P., Wang, Y., Fei, J., ... & Luo, G. (2024). Soil carbon storage and accessibility drive microbial carbon use efficiency by regulating microbial diversity and key taxa in intercropping ecosystems. Biology and Fertility of Soils, 60(3), 437-453.
35. Zhang, C., Shu, D., & Wei, G. (2021). Soybean cropping patterns affect trait-based microbial strategies by changing soil properties. Applied Soil Ecology, 167, 104095.
36. Bargaz, A., Noyce, G. L., Fulthorpe, R., Carlsson, G., Furze, J. R., Jensen, E. S., ... & Isaac, M. E. (2017). Species interactions enhance root allocation, microbial diversity and P acquisition in intercropped wheat and soybean under P deficiency. Applied Soil Ecology, 120, 179-188.
37. Yu, L., Luo, S., Gou, Y., Xu, X., & Wang, J. (2021). Structure of rhizospheric microbial community and N cycling functional gene shifts with reduced N input in sugarcane-soybean intercropping in South China. Agriculture, Ecosystems & Environment, 314, 107413.
38. Malviya, M. K., Solanki, M. K., Li, C. N., Wang, Z., Zeng, Y., Verma, K. K., ... & Li, Y. R. (2021). Sugarcane-legume intercropping can enrich the soil microbiome and plant growth. Frontiers in Sustainable Food Systems, 5, 606595.
39. Wang, T., Duan, Y., Lei, X., Cao, Y., Liu, L., Shang, X., ... & Zhu, X. (2023). Tea plantation intercropping legume improves soil ecosystem multifunctionality and tea quality by regulating rare bacterial taxa. Agronomy, 13(4), 1110.
40. Huang, Z., Cui, C., Cao, Y., Dai, J., Cheng, X., Hua, S., ... & Zhong, Z. (2022). Tea plant–legume intercropping simultaneously improves soil fertility and tea quality by changing Bacillus species composition. Horticulture research, 9, uhac046.
41. Sun, L., Dong, X., Wang, Y., Maker, G., Agarwal, M., & Ding, Z. (2022). Tea-soybean intercropping improves tea quality and nutrition uptake by inducing changes of rhizosphere bacterial communities. Microorganisms, 10(11), 2149.
42. Yang, Y., Feng, X., Hu, Y., & Zeng, Z. H. (2019). The diazotrophic community in oat rhizosphere: effects of legume intercropping and crop growth stage. Front. Agric. Sci. Eng, 6, 162-171.
43. Su, H., Lai, H., Gao, F., Zhang, R., Wu, S., Ge, F., ... & Yao, H. (2024). The proliferation of beneficial bacteria influences the soil C, N, and P cycling in the soybean–maize intercropping system. Environmental Science and Pollution Research, 31(17), 25688-25705.
44. Zhu, Y., Song, X., Wang, X., Chen, W., & Niu, X. (2022). The yield increase and land improvement effects of different sorghum/wild soybean intercropping patterns on reclaimed coastal salt pans. Journal of Soils and Sediments, 1-14.
45. Li, X., Sun, M., Zhang, H., Xu, N., & Sun, G. (2016). Use of mulberry–soybean intercropping in salt–alkali soil impacts the diversity of the soil bacterial community. Microb Biotechnol 9 (3): 293–304.
46. Lian, T., Mu, Y., Ma, Q., Cheng, Y., Gao, R., Cai, Z., ... & Nian, H. (2018). Use of sugarcane–soybean intercropping in acid soil impacts the structure of the soil fungal community. Scientific reports, 8(1), 14488.
47. Zhu Yihao, Li Jifeng, Dong Xiaoliang, Wang Songtao, Liu Zhiquan, Wu Zhenchen are the peaks (2024). The mixed planting mode of different types of reclaimed land in coastal areas has increased yield and improved the effect
48. Hong Dexing (2011). The effects of different intercropping crops on sugarcane growth and soil physicochemical properties (Doctoral dissertation, Fuzhou: Fujian Agriculture and Forestry University) [Hong D X. 2011. Effect of two different interplanting crops on the sugarcane growth and soil physical and chemical properties of cane fieldFuzhou: Fujian agriculture and Forestry University).
49. Dong Xiaogang, Tang Li, Zheng Yi&Deng Jiaqi (2015). The effect of root interaction on the number of rhizosphere microorganisms in different intercropping treatments of corn and soybean Journal of Yunnan Agricultural University (Natural Sciences), 30 (04), 624-628. doi:10.16211/j.issn.1004-390X(n).2015.04.022.
50. Zhou Li, Wang Yi, Li Dongbing, Liu Xiaoxue, Zeng Zebin, Wang Hongjiang& Liu Xuefeng (2022). The effects of different planting modes on soil fertility and soil bacterial community structure in mulberry orchards Southern Agricultural Journal, 53 (09), 2425-2434
51. Wei Chizhang, Nong Yuqin, Chen Yuanquan, Chen Haisheng, Wei Jinjian, Li Jinting& Qin Xiaomin (2018). The effect of tea tree/soybean intercropping on rhizosphere soil microbial community and enzyme activity Northwest Agricultural Journal, 27 (04), 537-544
52. Wei Jinjian, Qin Xiaomin, Nong Yuqin, Luo Yanfei, Lu Jinmei, Chen Yuanquan,&Wei Chizhang (2021). The effect of tea soybean intercropping on the metabolic diversity of soil microbial communities North China Agricultural Journal, 36(S01), 289-296.
53. Ren Qiyong (2023). Response of soybean yield and soil nitrification process to different farmland management methods (Master's thesis, Northwest A&F University) Master https://link.cnki.net/doi/10.27409/d.cnki.gxbnu.2023.000972 doi:10.27409/d.cnki.gxbnu.2023.000972.
54. Zhao Yiwen (2022). The role and mechanism of microbial community in regulating iron deficiency adaptability in soybean/maize intercropping system (Master's thesis, Northwest A&F University) Master https://link.cnki.net/doi/10.27409/d.cnki.gxbnu.2022.001528 doi:10.27409/d.cnki.gxbnu.2022.001528.
55. Zhang Chunfang (2021). The Impact Mechanism of Nitrogen Management Strategies on Soil Microbial Communities in Soybean Planting Systems (Doctoral Dissertation, Northwest A&F University)
56. Zhou Tao (2020). The Mechanism of Light Environment Regulation of High Efficiency Absorption and Utilization of Phosphorus in Corn and Soybean by Strip intercropping (Doctoral Dissertation, Sichuan Agricultural University)
57. Zhang Runzhi (2020). Research on the Mechanism of Nitrogen Fertilizer Regulating the Productivity, Nutrient Absorption, and Soil Microbial Effects of Corn/Soybean Intercropping Harbin: Northeast Agricultural University
58. Guo Li (2022). The impact of industrial hemp cultivation on soil microbial community structure and diversity in the black soil area of Northeast China (doctoral thesis, Northeast Forestry University) Dr https://link.cnki.net/doi/10.27009/d.cnki.gdblu.2022.001912 doi:10.27009/d.cnki.gdblu.2022.001912.
59. Bu Junyao (2020). Partial Study on the Promoting Effect of Intercropping Sugarcane and Soybean and Screening of Beneficial Rhizobacteria in the Rhizosphere (Master's Thesis, Guangxi University) Master https://link.cnki.net/doi/10.27034/d.cnki.ggxiu.2020.000890 doi:10.27034/d.cnki.ggxiu.2020.000890.
60. Hou Jian (2022). The effect of nitrogen supply level on bacterial and nutrient uptake and yield in rhizosphere soil of maize/soybean intercropping (Master's thesis, Northeast Agricultural University) Master https://link.cnki.net/doi/10.27010/d.cnki.gdbnu.2022.000209 doi:10.27010/d.cnki.gdbnu.2022.000209.
61. Li Ying (2021). The effect of nitrogen supply level on nitrogen uptake and rhizosphere nitrogen fixing bacterial diversity in maize/soybean intercropping system (Master's thesis, Northeast Agricultural University) Master https://link.cnki.net/doi/10.27010/d.cnki.gdbnu.2021.000374 doi:10.27010/d.cnki.gdbnu.2021.000374.
62. Chen Hongri (2018). The effects of intercropping in arid land on soil microorganisms, enzyme activity, and nutrient utilization efficiency (Master's thesis, Hunan Agricultural University) Master https://kns.cnki.net/kcms2/article/abstract?v=LzEBRIJt2Q258pMUg -cj2t5mlxv6WQTFOyqgbOh1tigCAmqOxiFLsryqbMIOA_crywEu6049UFpUXvwDoI8u_jOPDUJY1PTC_CASaOGWF35eNi6aqFnalkYffT1WZOIDOqUcSYyH62Hzqt-QXxjydBd42SyJHEbTIYf0FkzzBnl6UcG3EJgHzOUjJghEEbNe_hEi6ttdYzk=&uniplatform=NZKPT&language=CHS
63. Change frequently, Su Youbo, Fan Maopan, Wang Zilin, Zhao Jixia&Li Yongmei (2022). The rhizosphere microecological effects of maize soybean intercropping on red soil sloping farmland Journal of Shanxi Agricultural University (Natural Science Edition), 42 (02), 21-28. doi:10.13842/j.cnki.issn1671-8151.202111007.
64. Peng Donghai (2014). The effect of intercropping soybean on the physicochemical properties, bacterial and nitrogen fixing bacterial diversity of sugarcane rhizosphere soil (Master's thesis, Guangxi University) Master https://kns.cnki.net/kcms2/article/abstract?v=LzEBRIJt2Q0vKsIurXK4CqY2PH6gS0RpMhGuoTFsJ_C1b_MJublWF_JY_vxgiF18xDMVl9zB1W5GpO6OJqqCHP3FAFmqi_Bn42jDN8DONi8s -Bbu39XcbJ4N9BIc2kgl_AUCkqQ-kOK8X23_URZ3Tzx4Zg-g2qA4VInCZjgi68JEJMo7xJgLuszB5ajEdQcgRsK6DWbA_1Y=&uniplatform=NZKPT&language=CHS
65. Peng Donghai, Yang Jianbo, Li Jian, Xing Yongxiu, Qin Liudong, Yang Litao,&Li Yangrui (2014). The effect of intercropping soybean on the diversity of bacteria and nitrogen fixing bacteria in sugarcane rhizosphere soil Journal of Plant Ecology, 38 (9), 959-969
66. Xu Daixiang, Yang Jianfeng, Su Hang, Zhai Jianrong, Qi Cai, Zhao Longgang&Guo Yanjun (2023). The impact of metabolites in crop rhizosphere soil on microbial communities under intercropping mode Journal of Grassland Industry, 32 (11), 65-80
67. Fu Zhidan (2018). The impact of reduced nitrogen application on soil bacterial communities and greenhouse gas emissions in maize/soybean intercropping systems (Master's thesis, Sichuan Agricultural University)
68. Zhou Xianyu (2017). The effects of reduced nitrogen application and intercropping soybean on AMF infection and soybean nodulation in sweet corn and soybean (Master's thesis, South China Agricultural University)
69. Zhou Xianyu, Tang Yiling, Wang Zhiguo,&Wang Jianwu (2017). The effects of reduced nitrogen application and intercropping mode on AMF infection in sweet corn, soybean nodulation, and crop nitrogen and phosphorus uptakeping Journal of Ecological Agriculture (Chinese and English), 25 (8), 1139-1146
70. Ou Guangzhi, Zheng Liangbao, Liang Hong,&Zhou Lingyan (2024). Analysis of soil microbial community structure of soybean intercropping under kiwifruit forest Guangdong Agricultural Sciences, 51(1).
71. pingXin, Zhang Huihui, Yue Bingbing, Jin Weiwei, Xu Nan, Zhu Wenxu,&Sun Guangyu (2012). The effect of mulberry soybean intercropping on the diversity of carbon metabolism microorganisms in saline alkali soil Journal of Applied Ecology, 23 (07), 1825
72. Deng Wen, Hu Xingming, Yu Cui, Ye Chuhua, Li Yong, Xiong Chao,&Du Han (2015). The effect of intercropping soybean with mulberry trees on soil microbial diversity in mulberry orchards Sericulture Science, 41 (6), 997-1003
73. Qiao Yuejing, Guo Laichun, Ge Junyong, Liu Qi, Yang Zhenping,&Gao Zhiqiang (2020). The effect of intercropping oats and leguminous crops on soil enzyme activity and microbial biomass Journal of Gansu Agricultural University, 55 (3), 54-61
74. Jiang Qinfang (2023). The impact of interspecific interactions between maize and soybean intercropping on crops and soil (Master's thesis, Ningxia University) Master https://link.cnki.net/doi/10.27257/d.cnki.gnxhc.2023.001364 doi:10.27257/d.cnki.gnxhc.2023.001364.
75. Yan Li (2020). The effect of maize soybean intercropping on the composition of soybean root rot pathogens and the population structure of rhizosphere microorganisms (Master's thesis, Sichuan Agricultural University) Master https://link.cnki.net/doi/10.27345/d.cnki.gsnyu.2020.001358 doi:10.27345/d.cnki.gsnyu.2020.001358.
76. Song Yao, Zhou Sihao, Niu Hongjin, Zhang Xiaoxu, Huang Yali, Xing Mingzhen&Chen Xiaobo (2024). Analysis of bacterial community characteristics in maize root zone under maize soybean intercropping mode Environmental Science, 45 (08), 4894-4903. doi:10.13227/j.hjkx.202308270.
77. Gu Jiacheng, Wang Wenmin, Wang Zhen, Li Luhua, Jiang Guiju, Wang Jiaping,&Cheng Zhibo (2023). The effect of maize/soybean intercropping on the bioavailability of phosphorus and microbial community structure in rhizosphere soil Chinese Journal of Applied Ecology/Yingyong Shengtai Xuebao, 34(11).
78. Pi Yijun (2022). The effect and mechanism of maize soybean intercropping on the stability of organic carbon in yellow soil (Master's thesis, Guizhou University) Master https://link.cnki.net/doi/10.27047/d.cnki.ggudu.2022.000249 doi:10.27047/d.cnki.ggudu.2022.000249.
79. Wang Bingbing, Li Guangwen, Ma Kun, Liu Wenjuan, He Yaling, Jiang Qinfang&Mi Yang (2023). The impact of intercropping corn and soybean on soil fungal communities and crop productivity Agricultural scientific research, 44 (01), 6-14+21. doi:10.13907/j.cnki.nykxyj.2023.01.001.
80. Liu Li, Yang Jing,&Li Chengyun (2017). The effect of maize soybean intercropping on ammonia oxidizing microorganisms in maize rhizosphere Jiangsu Agricultural Journal, 33 (6), 1278-1287
81. Weng Qiaoyun, Huang Xinjun, Xu Hanlin, Liu Yao, Yuan Xiaofeng, Ma Hailian& Liu Yinghui (2021). The effects of maize/soybean intercropping mode on the yield, quality, soil nutrition, and rhizosphere microorganisms of silage maize Journal of Nuclear Agriculture, 35 (2), 462-470
82. Dai Zhenlin, Wang Yating, Yao Xiuying, Zhang Jinhao, Wang Yanfang, Yao Bo& Ji Guanghai (2020). The effect of intercropping mode between corn and soybean on the characteristics of soil microbial community in maize rhizosphere, maize yield and diseases Journal of Yunnan Agricultural University: Natural Science Edition, 35 (5), 756-764
83. Li Dongli (2022). Research on nitrogen absorption and rhizosphere soil nutrient and microbial characteristics in maize/soybean intercropping system (Master's thesis, Ningxia University) Master https://link.cnki.net/doi/10.27257/d.cnki.gnxhc.2022.001556 doi:10.27257/d.cnki.gnxhc.2022.001556.
84. Ma Haoran (2023). The effect of AMF on straw degradation and nutrient absorption in maize/soybean intercropping system (Master's thesis, Northeast Agricultural University) Master https://link.cnki.net/doi/10.27010/d.cnki.gdbnu.2023.000937 doi:10.27010/d.cnki.gdbnu.2023.000937.
85. Fan Yiwei, Xin Xiuli, Zhong Xinyue, Yang Jiao, Zhu Anning,&Zou Hongtao (2021). The effect of intercropping maize leguminous crops on soil enzyme activity and fungal community characteristics Soil, 53 (6), 1236-1243
86. Change frequently, Su Youbo, Fan Maopan, Zhao Jixia, Wang Zilin&Li Yongmei (2022). The effect of intercropping corn with soybean on the function and structure of rhizosphere microbial communities Journal of Yunnan Agricultural University (Natural Sciences), 37 (02), 336-343
87. Chen Yifu (2023). Analysis of rhizosphere microbiota and screening and construction of growth promoting microbial communities under monoculture and intercropping modes of maize and soybean (Master's thesis, Northwest A&F University) Master https://link.cnki.net/doi/10.27409/d.cnki.gxbnu.2023.000329 doi:10.27409/d.cnki.gxbnu.2023.000329.
88. Lin Weiwei, Li Na, Chen Lishan, Wu Zeyan, Lin Wenxiong,&Shen Lihua (2022). The impact of interspecific interactions between maize and soybean on the structure and diversity of rhizosphere bacterial communities Chinese Journal of Ecological Agriculture (Chinese and English), 30 (1), 26-37
89. Li Na (2016). The impact of interspecific interactions on the rhizosphere microecological characteristics of soybean and maize intercropping (Master's thesis, Fujian Agriculture and Forestry University) Master https://link.cnki.net/doi/10.27018/d.cnki.gfjnu.2016.000135 doi:10.27018/d.cnki.gfjnu.2016.000135.

**Supplementary Figures and Tables**

**Supplementary Figures**


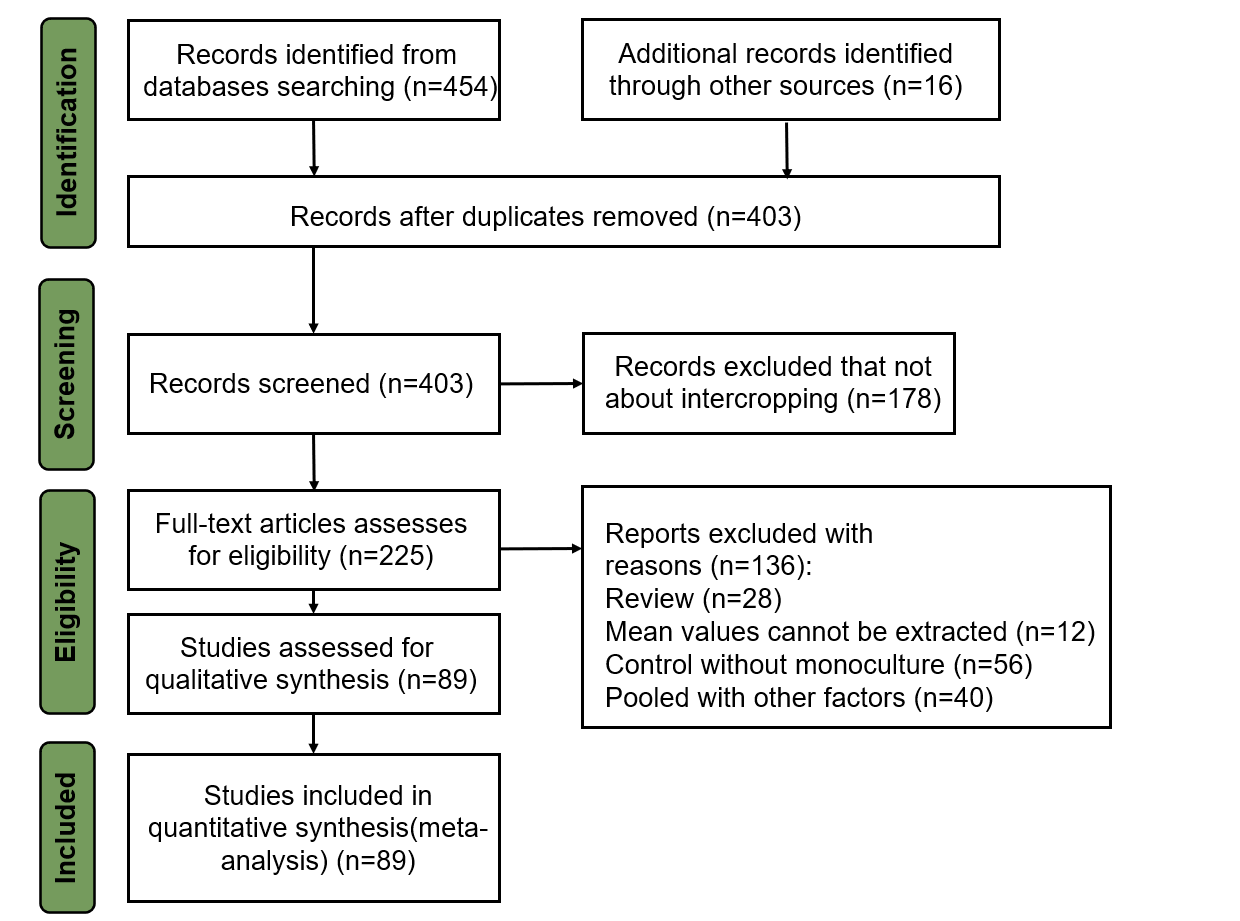


Fig. S1 PRISMA flow diagram showing the procedure used for selection of studies for synthesis


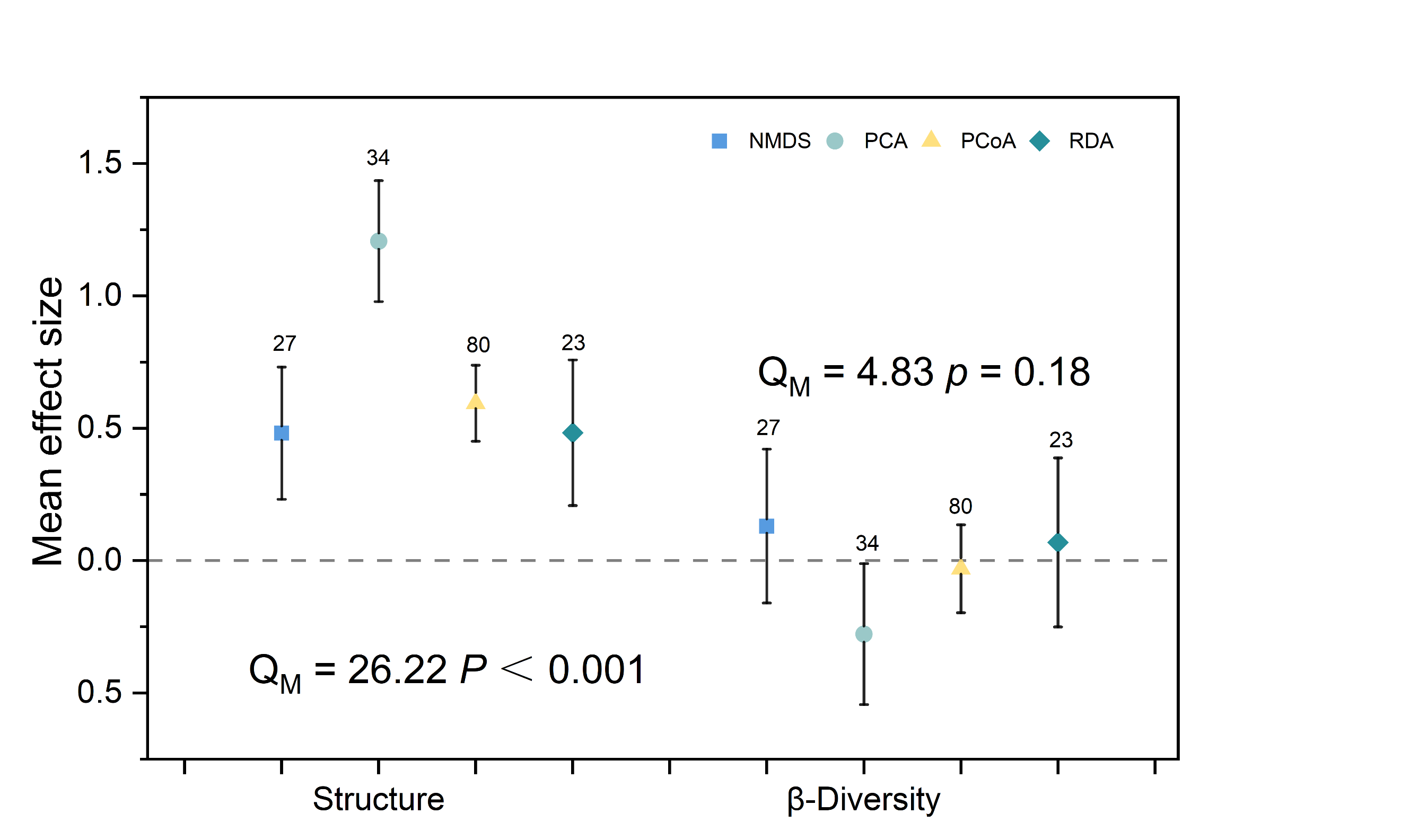


Fig. S2 Responses of microbial community parameters to soybean-based intercropping across different ordination analyses (NMDS: non-metric multidimensional scaling, PCA: principal component analysis, PCoA: principal correspondence analysis, RDA: redundancy analysis). The mean bar values are expressed as the mean effect size of each variable with 95% confidence intervals (CIs). The sample size of each variable is given at the top of the CIs. The significances of various moderators are tested by omnibus test (QM).


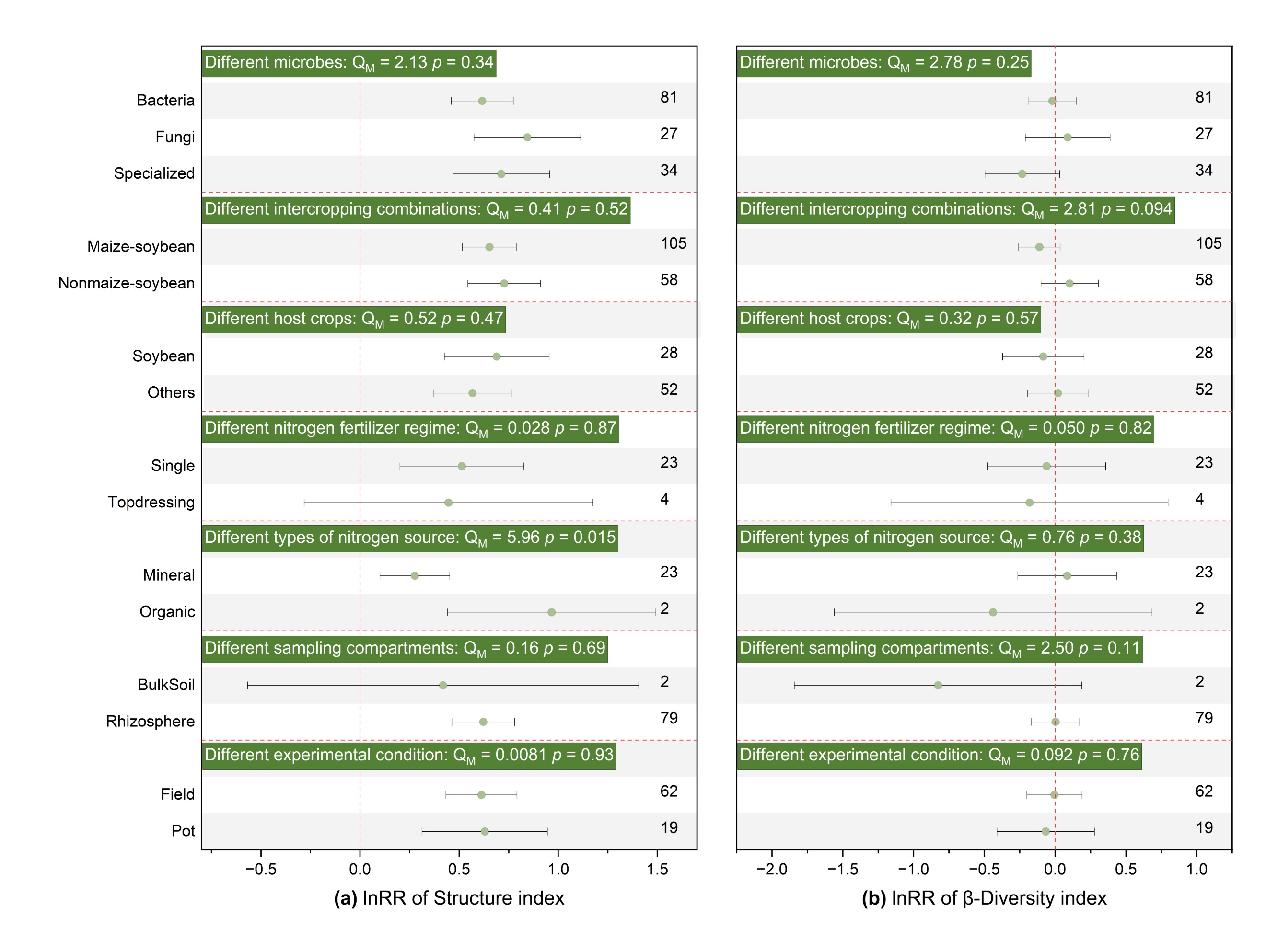


Fig. S3 Effects of soybean-based intercropping on soil microbial community Structure (a) and β-Diversity (b) parameters among different microbes, different intercropping combinations, different host crops, different nitrogen fertilizer regime, different types of nitrogen source, different sampling compartments, different experimental condition. The vertical dashed line was drawn at mean response ratio (RR) = 0. Error bars represent 95% confidence intervals (CIs), and the number on the y- axis indicate the number of observations. If 95% CI does not overlap the zero line, the effect of warming on a variable is considered significant If the 95% CI overlaps the zero line, the effect of warming is considered insignificant. A p < 0.05 indicates a significant difference between the subgroups. The significances of various moderators are tested by omnibus test (Q_M_).


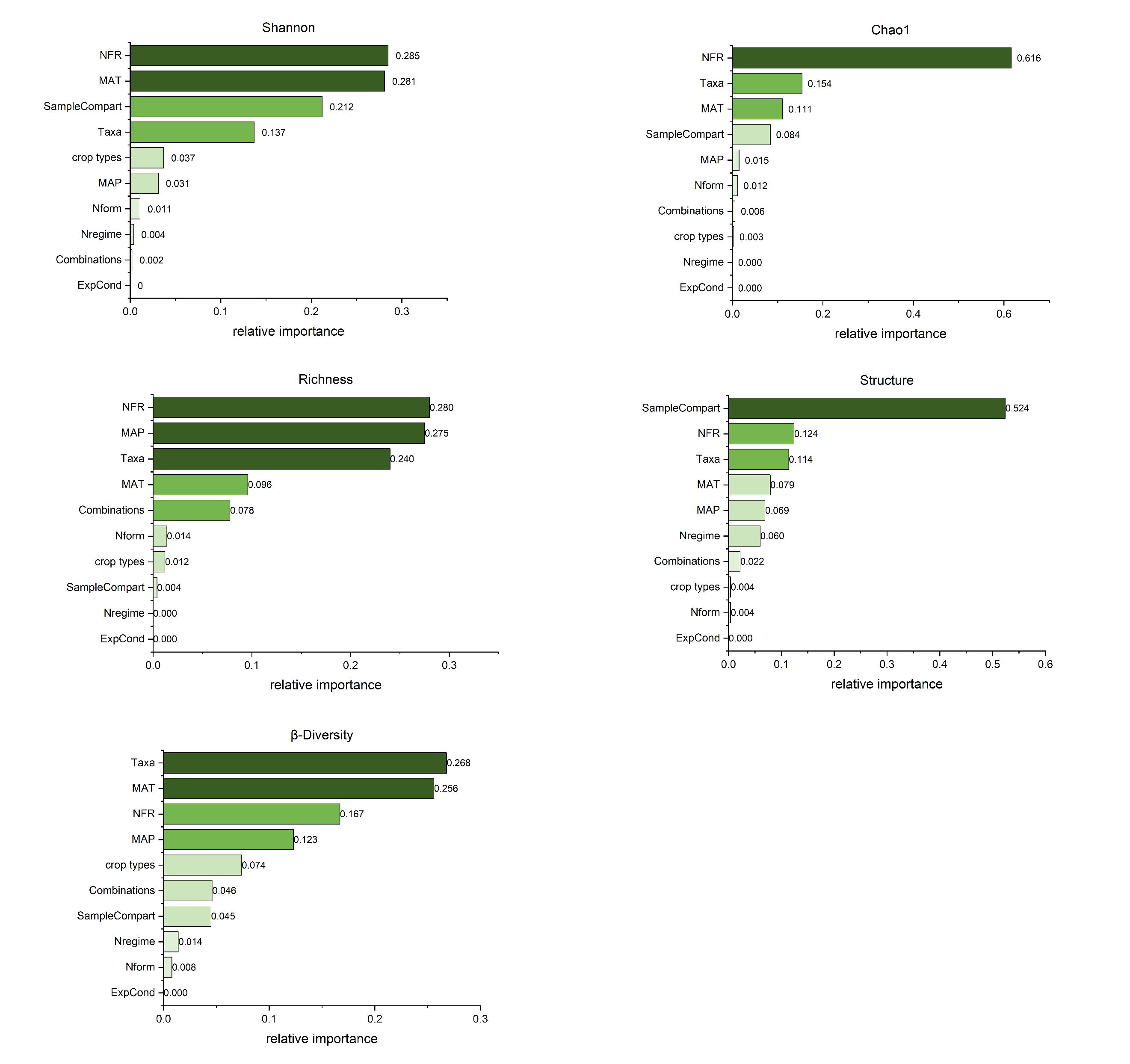


Fig. S4 Relative importance of factors affecting the effects of soybean-based intercropping on soil microbial community index namely Shannon, Chao1, richness, structure, and β-Diversity. MAP, mean annual precipitation; MAT, mean annual temperature; Taxa mean microbial species; NFR mean nitrogen application; host corp mean crop types; combinations mean species combinations; Samplecompart mean sampling compartments; Nform mean nitrogen sources; Nregime mean nitrogen fertilizer regime; ExpCond mean sampling compartments.


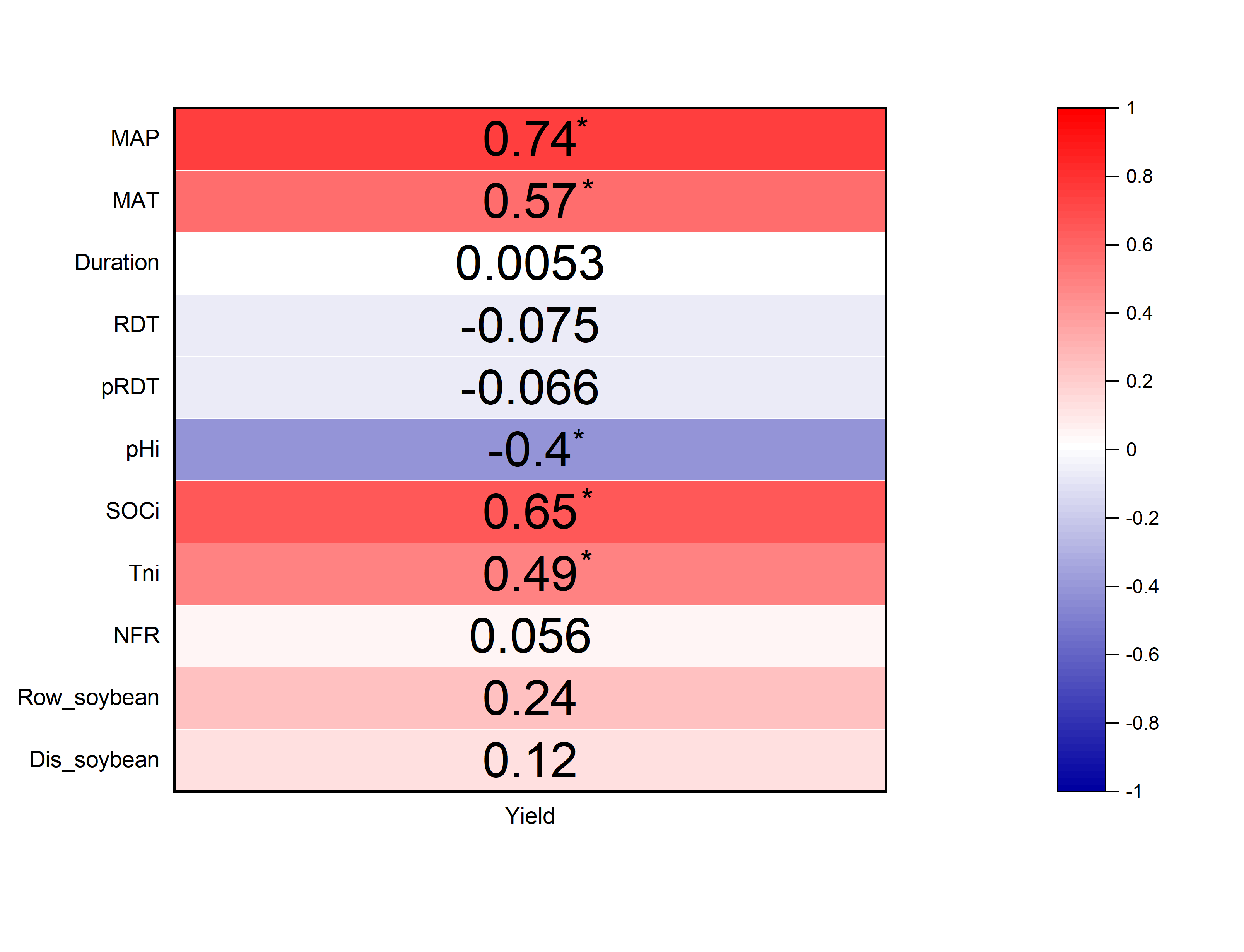
Fig. S5 The heatmap shows the correlation between soybean yield and environmental and management factors, with a color gradient denoting Pearson correlation coefficient. The numbers are correlation coefficients, “*” is significance mark. MAP, mean annual precipitation; MAT, mean annual temperature; Duration mean experimental period; RDT mean relative density total; pRDT mean density of intercropped soybeans relative to monocropped soybeans; pHi mean initial pH; SOCi mean initial SOC; Tni mean initial Total Nitrogen; NFR mean nitrogen application; Row_soybean mean soybean rows Dis_soybean mean soybean and other crop spacing.


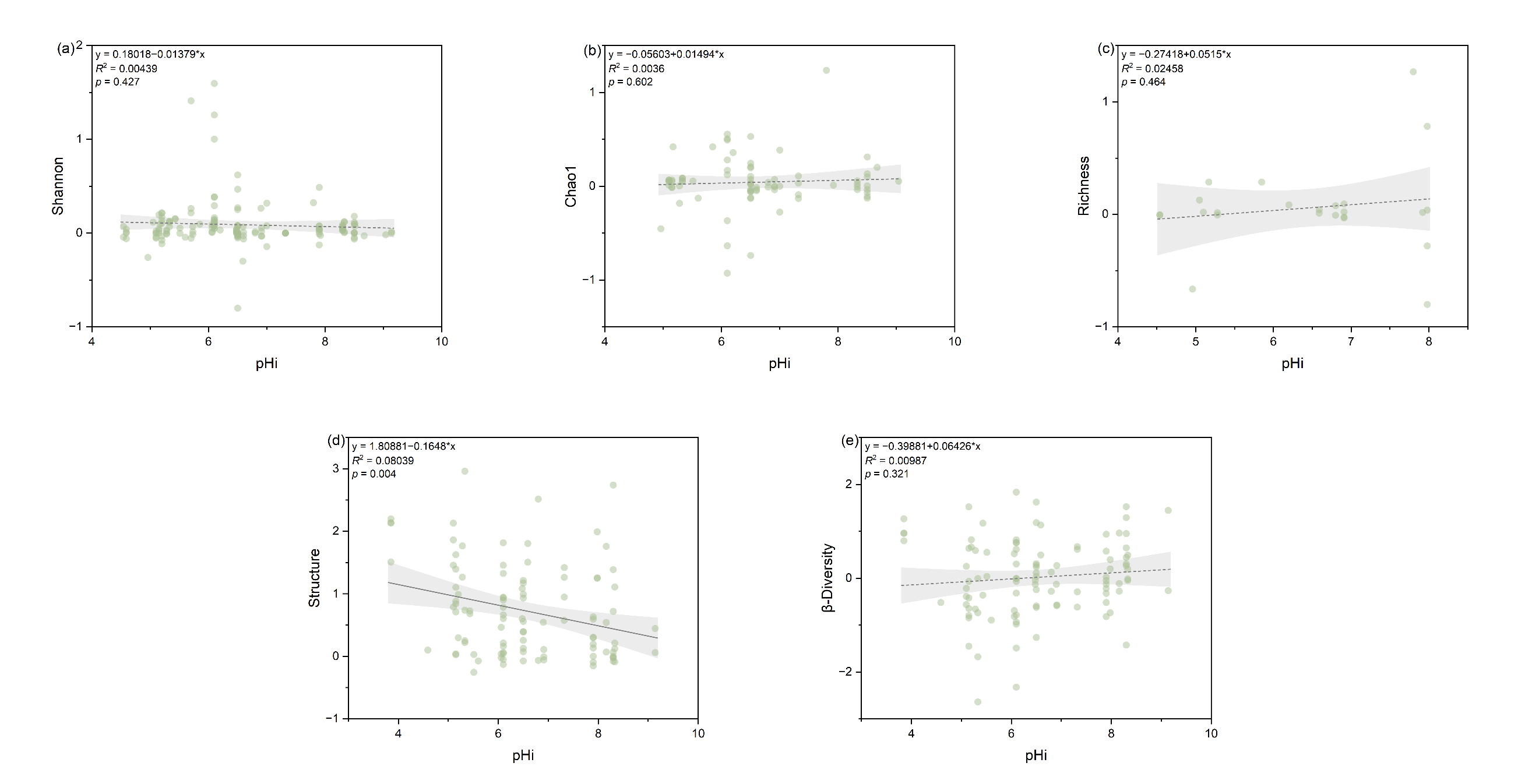


Fig. S6 Relationship between the response ratio of soil microbial community index, namely (a) Shannon, (b) Chao1, (c) richness, (d) structure, and (e) β-Diversity to initial soil pH as affected by soybean-based intercropping. The grey zone indicates 95% confidence intervals. The R^2^ and p-value of the models are noted.


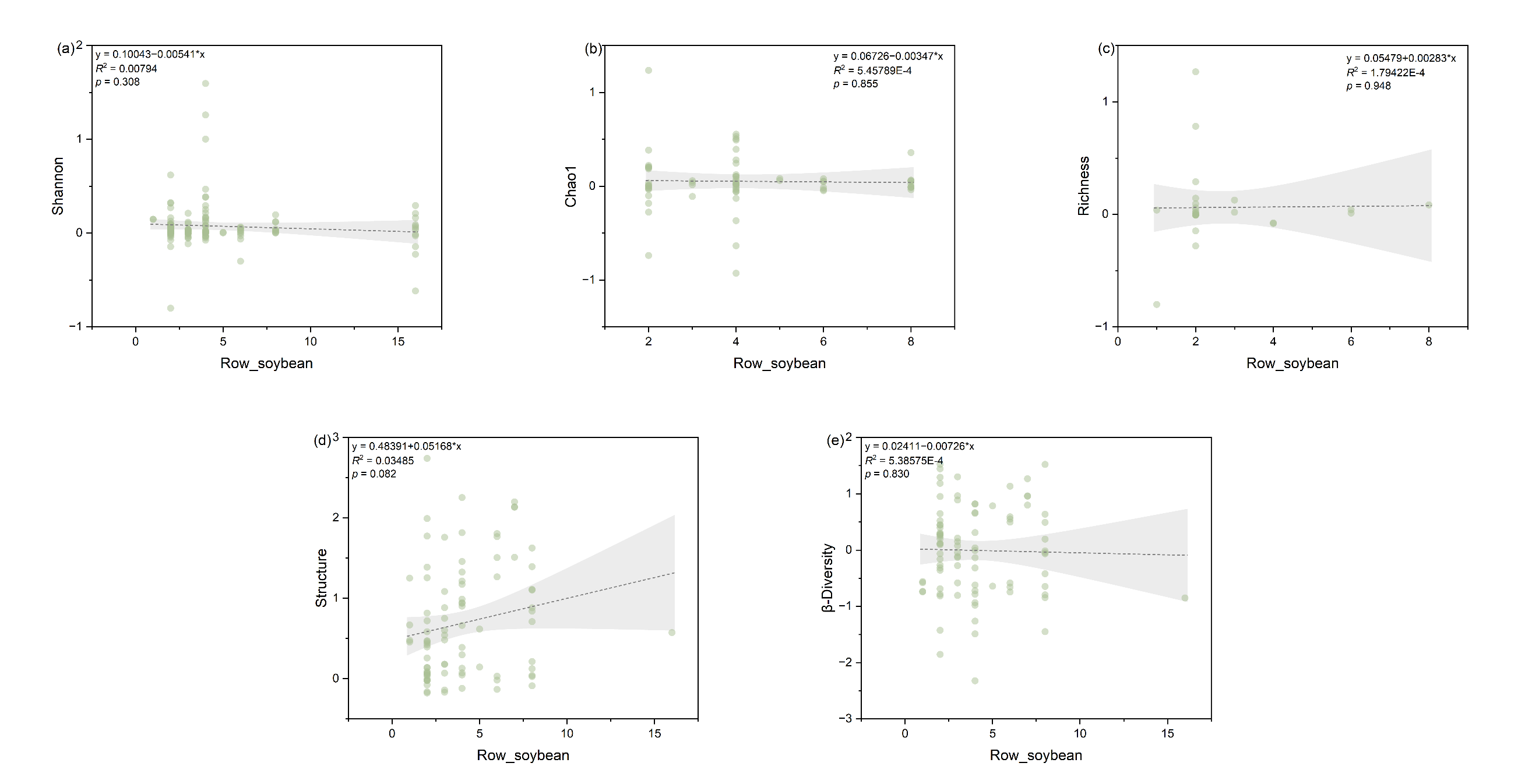


Fig. S7 Relationship between the response ratio of soil microbial community index, namely (a) Shannon, (b) Chao1, (c) richness, (d) structure, and (e) β-Diversity to the row of soybean as affected by soybean-based intercropping. The grey zone indicates 95% confidence intervals. The R^2^ and p-value of the models are noted.

**Supplementary tables**

Table S1 Results for the publication bias. k is the sample size for a variable. If the Fail-safe number is larger than 5n+10, the result is robust regardless of any potential publication bias. For Kendalls tau rank, the p value larger than 0.05 indicates the absence of publication bias.

| Variable | k | Fail-safe N | Kendall's p |
| --- | --- | --- | --- |
| yield | 71 | 124 | 0.3872 |
| Shannon | 212 | 40283 |  |
| Chao1 | 102 | 3592 |  |
| Richness | 55 | 841 |  |
| Structure  β-Diversity | 177  177 | 155125  1075 |  |

Table S2 Model selection for correlation analysis between microbial diversity index and yield. AIC means Akaike information criterion, BIC means Bayesian information criterion.

| Predictor | Best-fit Model | AIC | BIC | R^2^adj | p-val |
| --- | --- | --- | --- | --- | --- |
| Richness | Quadratic | -30.854 | -32.416 | 0.998 | <0.001 |
| Shannon | Quadratic | 38.190 | 46.071 | 0.068 | 0.053 |
| Structure | Quadratic | 40.780 | 46.885 | 0.124 | 0.049 |
| Chao1 | Linear | 25.970 | 29.504 | 0.091 | 0.082 |
| β-Diversity | Linear | 44.935 | 49.514 | -0.017 | 0.511 |
